# Dielectrophoresis: Developments and applications from 2010 to 2020

**DOI:** 10.1002/elps.202000156

**Published:** 2020-12-28

**Authors:** Benjamin Sarno, Daniel Heineck, Michael J. Heller, Stuart D. Ibsen

**Affiliations:** ^1^ Oregon Health and Science University–The Knight Cancer Institute's Cancer Early Detection Advanced Research Center Portland OR USA; ^2^ University of California San Diego–Nanoengineering La Jolla CA USA; ^3^ Oregon Health and Science University–Biomedical Engineering Portland OR USA

**Keywords:** Biotechnology, Cell separation, Dielectrophoresis, Nanofabrication, Nanotechnology

## Abstract

The 20th century has seen tremendous innovation of dielectrophoresis (DEP) technologies, with applications being developed in areas ranging from industrial processing to micro‐ and nanoscale biotechnology. From 2010 to present day, there have been 981 publications about DEP. Of over 2600 DEP patents held by the United States Patent and Trademark Office, 106 were filed in 2019 alone. This review focuses on DEP‐based technologies and application developments between 2010 and 2020, with an aim to highlight the progress and to identify potential areas for future research. A major trend over the last 10 years has been the use of DEP techniques for biological and clinical applications. It has been used in various forms on a diverse array of biologically derived molecules and particles to manipulate and study them including proteins, exosomes, bacteria, yeast, stem cells, cancer cells, and blood cells. DEP has also been used to manipulate nano‐ and micron‐sized particles in order to fabricate different structures. The next 10 years are likely to see the increase in DEP‐related patent applications begin to result in a greater level of technology commercialization. Also during this time, innovations in DEP technology will likely be leveraged to continue the existing trend to further biological and medical‐focused applications as well as applications in microfabrication. As a tool leveraged by engineering and imaginative scientific design, DEP offers unique capabilities to manipulate small particles in precise ways that can help solve problems and enable scientific inquiry that cannot be addressed using conventional methods.

**Abbreviations**
AC: alternating current
AFM: atomic force microscopy
BPD: benign pancreatic disease
cDEP: contactless DEP
CTC: circulating tumor cell
DENV: dengue virus
GFAP: glial fibrillary acidic protein
DC: direct current
DEP: dielectrophoresis
EV: extracellular vesicles
fDEP: fluidic DEP
iDEP: insulator‐based DEP
ITO: indium tin oxide
PDAC: pancreatic ductal adenocarcinoma
SWCNT: single‐walled carbon nanotube

## 1 Introduction

The study and application of the electrokinetic phenomena of dielectrophoresis (DEP) has been maintaining momentum over the 2010–2020 decade. Starting in the 1920s, Hatschek and Thorne described DEP in essence and H.S. Hatfield developed a dielectric separation technique patent and built a pilot plant to separate cassiterite from quartz [1, 2]. In 1951, the term DEP was first coined by H.A. Pohl in his paper, *“The Motion and Precipitation of Suspensoids in Divergent Electric Fields”* [3]. A Google Scholar search for “dielectrophoresis” produces nearly 29 700 entries since 1951. Also notable is the commercial application of dielectrophoretic‐based technologies, with over 2600 patents held by The United States Patent and trademark Office (USPTO), with 106 being filed in 2019 alone.

**Figure 1:**
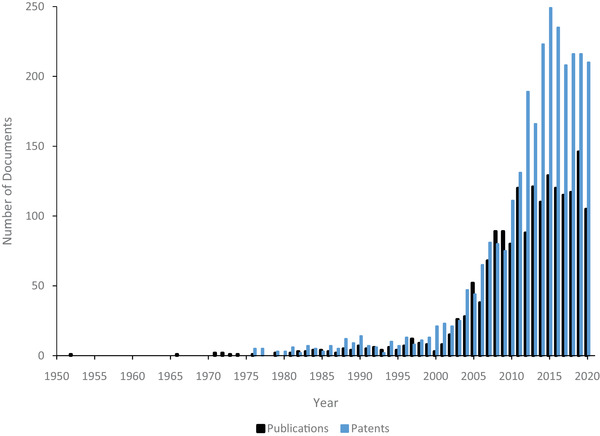
An overlaid distribution of the number of patents and publications in the DEP field over time. The graph starts with Pohl's 1952 industrial process paper and goes up to March 2020 [3]. Statistics were taken from a PubMed, open source, date restricted search for “*Dielectrophoresis*” and a date restricted search of the USPTO database for “*Dielectrophoresis*.” There was a large rise in annual patents and publications between 2000 and 2010, which has maintained relatively constant through this past decade.

**Figure 2:**
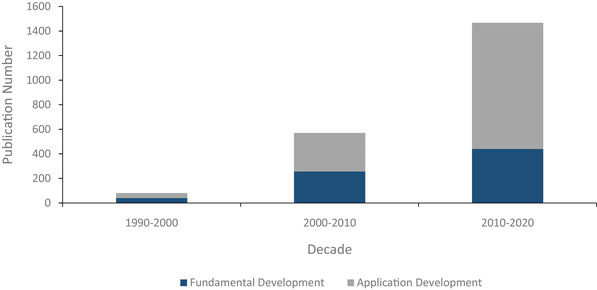
PubMed publication distribution by decade. The categorized distribution of publications across three decades. Publication data from PubMed shows a clear shift from fundamental DEP technology development to applications. Nearly 50% of the DEP publications were application based in the 1990s, rising to approximately 55% between 2000 and 2009, and to 70% of the publications for the 2010–2020 decade. The *x*‐axis is divided into the three respective decades, and the *y*‐axis represents single publications. The split columns of dark blue and gray portray the shifting ratio as well as the increase in volume of publications in the field across the three decades.

**Figure** **1** shows the distribution of publications and United States patent holdings between 2010 and May 2020 from PubMed and The United States Patent Office and Trademark websites, respectively. The bulk of the patents and publications before 2010 were discoveries of range and potential, whereas much of translational progress in DEP has occurred in the past 10 years. Yearly publications steadily increased between 2000 and 2010, while the annual publication content across 2010–2020 remained relatively constant, with two spikes in 2015 and 2019, respectively, being a 21% and a 37% increase over the decade's average. An open source Pubmed search for “Dielectrophoresis” produces a total of 112 results from 1951 to 2000, followed by 613 from the 2000s, and 981 publications from 2010 to mid‐2020. **Figure** **2** shows the categorized distribution of publications across all three decades. There has been a shift from fundamental DEP technology development to more of an applications focus, with a nearly 20% increase in application patents across the two decades. This change in publication distribution shows a maturity in DEP fundamentals, while new applications remain abundant.

Notably, biological applications of DEP have been gaining traction as well over the 2010–2020 decade. Pohl and Hawk were the first to show the separation of live and dead yeast cells in 1966 [4]. Since then, biotechnology applications have led the DEP field for the past 30 years. In fact, 20 of the latest 21 United States patents for DEP are related to biotech applications.

This review focuses on work of the past decade to outline the progress of DEP‐based biotechnology and nanotechnology platforms with emphasis on applications. Specific DEP topics include cell sorting devices, virus and bacteriophage manipulation, blood biomolecule isolation, nanoparticle DEP, DEP nanofabrication, and liquid DEP. Other recommended, DEP‐centric reviews focused on different time periods, applications, theory, and technological aspects are widely available [2, 5, 6, 7, 8, 9, 10].

## 2 Background/theory

It is important to begin with the basic principles of DEP, as they provide fundamental context for all the advances in computer modeling, fabrication techniques, and materials science that have been realized in the past decade. DEP is the movement of particles through a medium in response to a nonuniform electric field. Classic, time‐averaged, DEP theory is based on applying Maxwell's equations to a homogeneous, single particle in a semi‐infinite medium where the electric field gradient across the particle is small. Within this approximation, Equation 1 can be used to model the time‐averaged DEP force, with positive values signifying a force toward an increasing electric field gradient.

(1) F⇀DEP=2πεmR3ReCM*∇ERMS2

The terms are as follows: $\varepsilon_{m}$ is the absolute permittivity of the theoretically infinite and homogeneous medium, CM is the frequency‐dependent Clausius–Mossotti factor relating to the polarizability of the particle and medium, R is the particle radius, and $E_{\mathrm{RMS}}$ is the root‐mean‐square of the electrical field. Unlike electrophoresis, which relies on a particle's charge, the DEP force affects any material that can be differentially polarized relative to its suspending medium. The Clausius–Mossotti factor, shown in Equation 2, is a unitless number that quantifies the interaction between the particle and medium in terms of their respective complex polarizabilities, and determines the DEP force direction. It is written in its complex form as:

(2) $$C\;M^{*}=\left(\frac{\varepsilon_{p}^{*}-\varepsilon_{m}^{*}}{\varepsilon_{p}^{*}+2\varepsilon_{m}^{*}}\right)\;\;\mathrm{or}\;C\;M^{*}=\left(\frac{\sigma_{p}^{*}-\sigma_{m}^{*}}{\sigma_{p}^{*}+2\sigma_{m}^{*}}\right)$$

where both the complex permittivity of the particle ($\varepsilon_{p}^{*})$ and the media ($\varepsilon_{m}^{*})$ and the complex conductivity of the particle$\;(\sigma_{p}^{*})$ and of the media $\left(\sigma_{m}^{*}\right)$ are frequency dependent [11, 12]. The specific Clausius–Mossotti factor versus frequency relationship depends on the respective properties of the constituents of the system. Conductivity terms tend to dominate the CM relation below 50–100 kHz and permittivity terms above ≈1 MHz, with a transition region in between. For a given electric field gradient, DEP may exist at all electric field frequencies other than the crossover frequency, that is, Re(CM)=0, where there is no differential polarizability between the particle and the medium. It also marks a transition between positive and negative CM relation frequency regimes, so frequency selection provides an additional lever for particle manipulation. For example, mammalian cells in aqueous buffers have negative‐to‐positive crossover frequencies in the range of 1–20 MHz, regardless of the medium's conductivity. Other biological samples like bacteria, many phages, various vesicles, complex protein structures, and nucleic acids largely exhibit a positive CM relation in the conductivity‐dominated frequency range, even in high conductance media such as phosphate buffered saline (PBS) or plasma. It is important to note that this is a simplified model of DEP that describes the first‐order driving terms. To accurately model more complex particles that are not uniformly homogeneous, such as cells, multishell dielectric models have been developed [13, 14, 15]. Further theoretical modeling and in depth consideration of these classical physical effects are available in numerous papers that approach these issues from different engineering, materials science, and physics‐based perspectives [16, 17, 18, 19, 20]. Similarly, another resource is myDEP, which is a computational toolkit that allows the user to model various particles, including multishell cellular particles, and particle‐field interactions [21].

As alluded to above, biological particles are predominately conductive, largely due to maintaining a static charge. This means that in a direct current (DC) field, electrophoresis will dominate over any translational DEP effect. An alternating current (AC) signal is needed to temporally average out the electrophoretic force on the particle. It also mitigates against ion screening of the particle, electrolysis of the media, and electroosmotic flow. Frequency choice may also control the electrochemistry in some cases [22]. Additional factors may affect the electrical properties of the constituents as well, such as the local ion concentration in the medium or temperature of the system. For example, the relative dielectric constant of pure water is 80 at 25°C and approximately 65 at 60°C [23]. Joule heating and dielectric loss are always present in the medium but become especially significant under high voltage operation, and/or in mediums with high ion concentrations, for example, many biological fluids [24]. Depending on operating parameters, system temperatures can rise by tens of degrees Celsius with local temperatures much higher, which dramatically changes the material properties in the affected regions. An additional concern to most biologically relevant high conductance buffers is the amount of electrochemical activity occurring at the electrode–buffer interface, which, in addition to heat, may degrade both the electrode and biological products captured nearby.

The system's electric field gradient, $\nabla |E^{2}|$, is heavily dependent on device geometry, providing an engineering opportunity to control particle trajectories. Large electric field gradients can be formed through small conductive electrodes, either directly energized or at a floating potential, in the region of interrogation, or through insulator‐based DEP (iDEP), where the electrodes are placed far away from structures formed from electrically insulating materials [25, 26]. These insulating structures locally pinch the electric field to create regions with large field gradients, which can function as independent capture or trapping regions [27]. This serves to separate the harmful electrochemistry occurring at the driven electrode from the sample, making iDEP preferable for high‐conductance biological samples. Reduced demand for electrode materials, often noble metals, helps to reduce cost and improve access to these cutting‐edge technologies [25, 28]. Since the electric field needs to concentrate far away from the electrodes themselves, iDEP is usually performed at much higher voltages compared to direct electrode‐based DEP.

Due to the relatively low force magnitudes, DEP is largely used in quasi‐static fluid conditions, where the particles collect into either the high or low field gradient regions, according to the conditions and device geometry. However, bulk fluid flow can play an important role in DEP applications involving flow‐fractionation based particle separations where DEP is used to steer particles into desired flow streams, including the use of traveling wave DEP [29], which requires careful phasing across multiple interdigitated electrodes [28, 30, 31, 32, 33]. Traveling wave DEP holds promise in spectroscopic analysis of particles and cell identification, summarized by Turcan and Olariu [34].

Even within the “small, homogenous, spherical particle” assumption generally applied to biological samples, particle size is a driving force in achieving particle selectivity [35]. Fluid drag is proportional to cross section, that is, $F_{\mathrm{drag}}\propto r_{\mathrm{particle}}^{2}$, whereas $F_{\mathrm{DEP}}\propto r_{\mathrm{particle}}^{3}$, meaning that the drag force resulting from DEP‐driven particle movement will become increasingly close to the DEP force as radius decreases. It also means that for two particles that vary only in size, the smaller particle will see an increased contribution from drag force (external fluid forces, e.g., Brownian motion or advection) relative to the contribution from the DEP force, which affects particle motion. The contribution from the drag force becomes more significant as particles shrink below ≈50 nm, ultimately limiting the size of particles that can be collected or trapped by DEP [17, 36]. The magnitude of the actual transient effects on cells and other large particles can be approximated from the creeping‐flow limit of Navier–Stokes hydrodynamic flow equations under static conditions, but not in flow‐focusing systems where the Reynolds numbers are generally in the range of 1–10 [17]. Despite this limitation, DEP's applicability across a wide range particle sizes (≈10^–8^–10^–3^ m), materials, media, and dielectric properties lends to its functionality [36, 37, 38, 39].

## 3 Biological applications

One of the unique aspects of DEP technology development over the last 10 years has been its versatility and wide applicability to address different biological questions. Collecting and concentrating molecules and biologically derived particles ranging from cancer‐derived nanoparticles all the way to whole cells has important potential in allowing researchers to ask questions they have never been able to before. When bounded by certain electrochemical constraints, the phenomenon allows for manipulation of biological particles without long‐term damage or deterioration of the material of interest [40]. The following highlights some of the research that demonstrates the types of applications DEP can contribute to in unique ways.

### 3.1 Molecular DEP

Although DEP is often applied to particles ranging in size from tens of nanometers all the way to cell‐sized structures, DEP can achieve collection on the molecular level under the right electrode geometries and applied electric field intensities. As discussed in the theory section, as particles decrease in size, proportionally larger electric field gradients are required for DEP manipulation and immobilization, which can be achieved through increasing applied voltage and reducing device dimensions.

Laux et al. fabricated a system for trapping the enzyme horseradish peroxidase using a standard 0.25 μm complementary metal oxide semiconductor device integrated with 500 nm diameter tungsten cylinders, which were arrayed on a 2 μm pitch [41]. Optimal enzyme capture occurred at 10 kHz. Bubble formation due to electrolysis occurred and systems were overwhelmed with bubbling below 5 kHz, and fluid motion began to occur above 1 MHz. Enzymatic activity of trapped horseradish peroxidase was demonstrated by quantifying oxidation of dihydrorhodamine‐123 to fluorescent rhodamine 123 (Rh123) by horseradish peroxidase and hydrogen peroxide. This confirmed the DEP device's ability to immobilize submicron particles with a modest enough voltage to maintain its natural functionality.

**Figure 3:**
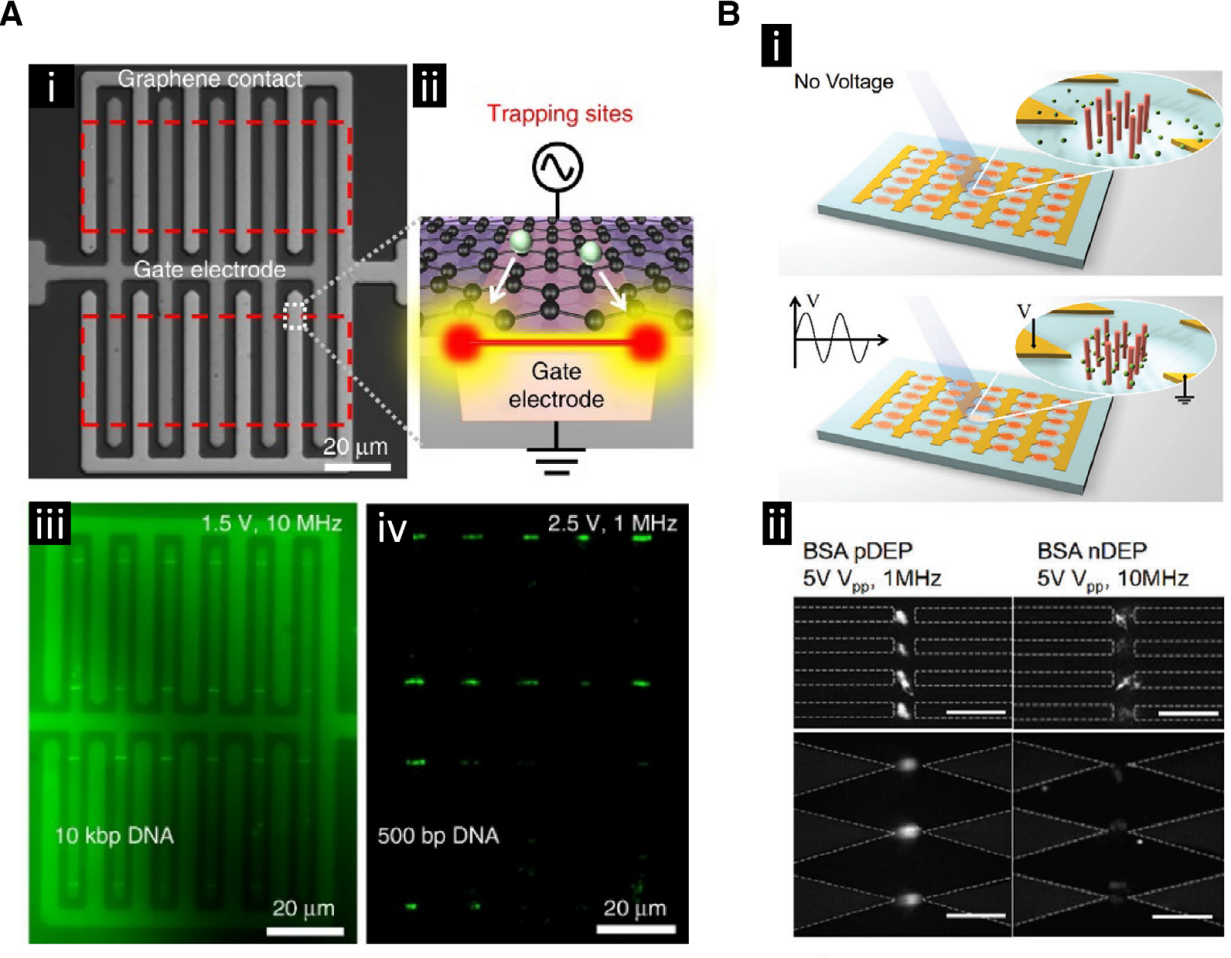
The use of DEP to collect and detect molecules. (**A)** Collection of DNA. **(i)** Bright field image of the interdigitated electrode array design. **(ii)** Schematic representation of the collection zone at the edge of the graphene electrode. **(iii)** Collection of fluorescently labeled 10 kbp DNA. **(iv)** Collection of 500 bp DNA. Reproduced under the terms of the Creative Commons Attribution 4.0 International Public license. [42] Copyright 2017, The Authors. Published by Springer Nature. **(B)** iDEP strategy that uses integrated pillars to enhance the electric field strength for collection of molecules. **(i)** Schematic representation of the chip design with pillars in the insulator gaps showing the enhanced collection of molecules around the pillars. **(ii)** Fluorescent images showing the frequency dependency of bovine serum albumin (BSA) protein capture using two different iDEP chip designs. Scale bars are 20 μm. Adapted with permission from [43]. Copyright 2018, WILEY‐VCH.

Barik et al. developed a graphene‐edge DEP trapping platform that was capable of reversibly trapping nanoparticles and biomolecules with nanoscale precision (Fig. 3A) [42]. Back‐gated graphene devices with an 8 nm thick HfO_2_ dielectric were fabricated using standard nanofabrication processes. Devices were patterned such that the top and bottom metal contacts formed an interdigitated pattern. Trapping sites were defined by the width of the gate electrode and the vertical gap between the two electrodes. This sub‐10 nm gap generated DEP forces that could be 10X greater than forces produced by metal electrodes. The graphene device demonstrated an ability to manipulate 10 kbp and 500 bp DNA, prestained with YOYO‐1 dye, at a 10 pM concentration. The device was also shown to enable capture of 190 nm, fluorescently labeled, polystyrene beads and 40 nm diamond nanoparticles with nitrogen vacancy centers, which acted as quantum emitters and thus allowed for fluorescent detection. The spatial confinement, due to the small device dimensions, could enable low‐concentration assays and the ability to examine localized molecular interactions. Edge‐trapping DEP devices can be integrated with graphene nanoresonators or tapered nanotips, which could harness both DEP trapping and mid‐IR spectroscopy of low‐concentration biomolecules. Additionally, quantum emitters can be positioned with nanometer resolution to build photonic circuits or single‐photon source arrays.

Cao et al. developed an iDEP platform capable of rapidly enriching proteins for a high‐sensitivity immunoassay (Fig. 3B) [43]. The device was composed of a structure with 5 μm interelectrode gaps, where these gaps were filled with 100 nm nanorods on a 100 nm inter‐rod spacing. Iterations of the device were made with both sawtooth and castellated electrode geometries, and both SiO_2_‐coated Ag nanorods and pure SiO_2_ nanorods were deposited into the gaps. The behavior of the two different types of rods under an externally applied voltage was modeled using COMSOL software. The simulations showed that the SiO_2_‐coated Ag nanorods created a significantly higher electric field intensity compared to the pure SiO_2_ nanorods. Bovine serum albumin protein labeled with Alexa 488 exhibited DEP with respect to the nanorod array, with positive DEP capture occurring within 10 s using a signal at 1 MHz. The fluorescent intensity began to plateau with increased time, and negative DEP was observed at 10 MHz. The devices with SiO_2_‐coated Ag nanorods displayed an enrichment factor nearly ten times greater than the SiO_2_ nanorods, and sawtooth electrodes slightly outperformed the castellated electrodes devices. The device constructed with both sawtooth electrodes and SiO_2_‐coated Ag nanorods had an average enrichment rate of 180‐fold per second, the highest reported enrichment technique through 2018. Goat anti‐mouse IgG antibodies were then functionalized to the nanorod surface, and mouse antigens were captured via positive DEP (1 MHz), with an Alexa 488‐conjugated rabbit anti‐mouse IgG antibody used as a fluorescent probe. The device with sawtooth electrodes and SiO_2‐_coated Ag nanorods achieved a lower limit of detection (LOD) of 275.3 fg/mL.

Sanghavi et al. showed the detection of neuropeptide Y and orexin A using a frequency‐selective DEP‐based technique to preconcentrate the proteins in a constricted flow nanochannel [44]. Negative DEP was applied to the system for 10 s at 3 MHz, with a DC bias to trap the proteins away from the lateral insulator constrictions, which ran across the flow channel, and on to graphene‐modified electrodes, which were aligned orthogonally to the nanochannel near the lateral constriction. The small size of the channels allowed sub‐nanoliter sample volumes to be analyzed. This enabled the electrochemical detection of neuropeptide Y down to 4 pM and orexin A down to 22 pM. Chaurey et al. showed the trapping and concentration of single‐stranded DNA using a similar device design [45].

**Figure 4:**
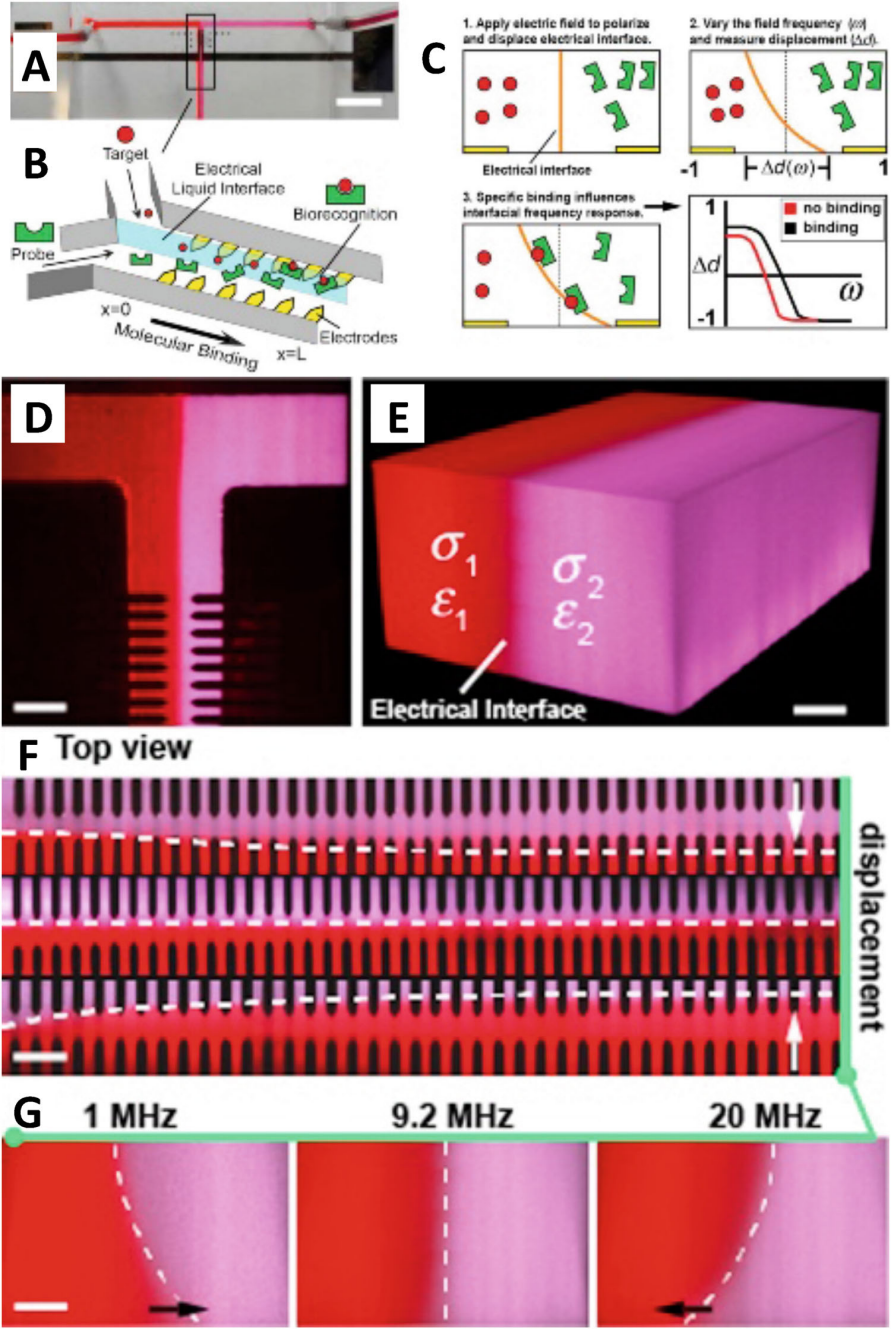
Use of DEP to detect molecular interactions. (**A)** A liquid interface is created using a T‐channel microfluidic device. Scale bar 5.0 mm. (**B)** Integrated electrodes in the channel influenced the electrical liquid interface to create the interfacial electrokinetic transduction (IET) sensor. (**C)** The applied electric field polarized the interface between the two liquids and displaced it a distance Δ*d*. This distance was a function of the field frequency. If binding occurred between the target and the probe at the fluid interface, then the frequency dependency of the interface displacement would change, which indicated target detection. **(D)** The two fluorescently labeled fluids are shown in the T junction forming a liquid interface. Scale bar 50 μm. (**E)** A 3D reconstruction of the fluid interface using confocal imaging. Scale bar 20 μm. (**F)** As the fluid passed by the electrode array its displacement could be measured as a function of the applied frequency. Scale bar 50 μm. (**G)** A side view of the fluid profile as a function of the applied frequency using confocal microscopy. Scale bar 20 μm. Reprinted from [46]. Copyright 2015, with permission from Elsevier.

Mavrogiannis et al. demonstrated a different type of DEP‐based molecule detection that used an electrical liquid interface generated with microfluidics (Fig. 4) [46]. This label‐free technique, called fluidic DEP, used two different fluids that ran alongside one another through a channel and through an externally applied AC electric field with very little mixing. The target biomolecule bound to the liquid interface, which changed the electrical conductivity of the interface. This change caused the interface to shift spatially under the influence of the AC electric field making this an interfacial electrokinetic transduction method. This shift was detected using fluorescence microscopy. They showed that this technique can be used to detect low femtomolar concentrations of avidin protein from within a 5 mg/mL background of serum albumin. The system can be adapted to detect other proteins as well. The phenomena of fluidic DEP can also be used to enhance mixing between two co‐flowing fluids that have different electrical properties by taking advantage of the interface deflection to induce chaotic advection, thereby reducing mixing time [47, 48].

It has been demonstrated that a wide range of different DEP device designs are capable of trapping molecules including both insulator‐based and electrode‐based devices. One thing they have in common is cleverly designed geometries that help to increase the electric field intensity and thereby the DEP force to levels capable of trapping molecules. The ability to trap enzymes and maintain their function is a particularly impressive and useful ability. The enzymatic activation of substrate by the trapped enzyme can be used to get a signal amplification effect that allows smaller amounts of the enzyme to be detected than could be achieved by direct detection of the enzyme itself. Future developments that would be advantageous include trapping molecules in high conductance solutions that approach the salinity of biological fluids, which would simplify the preparation of collected biological samples and would prevent dilution of the samples keeping the targets as concentrated as possible.

### 3.2 Viruses

Viruses constitute an important set of biologically based particles both for their use in biotechnology as DNA transfection agents and for their role in infectious disease. Detecting and recovering viruses from various biofluids is challenging using conventional methods, especially if the viral particles are dilute. DEP has a unique capability to contribute to the collection, detection, and study of virus particles due to its ability to concentrate them in a label‐free way. This could open up future possibilities of using DEP‐based technology for pathogen detection in the clinical setting.

Ding et al. designed a gradient iDEP device that employed sawtooth‐patterned insulating structures that incrementally decreased in pitch to concentrate the *Sindbis* virus [49]. The iDEP devices utilized gap distances that decreased from 30 to 3 μm by increasing the side length and width of the PDMS triangles. This geometry created a characteristic electric field determined by device architecture, which increased linearly throughout the channel. Fluorescently labeled viral particles showed a significant level of capture above a voltage threshold. The device showed a 2x and 6x increase in *Sindbis* viral concentration as compared to other methods used to detect the virus, including immunofluorescent techniques, ELISAs, western blots, and green fluorescent protein binding. The device ultimately demonstrated the direct capture and detection of viruses.

Iswardy et al. fabricated a DEP microfluidic device for immunofluorescent detection of the dengue virus (DENV) [50]. Silica beads (1 μm) were modified with mouse anti‐flavivirus mAbs capable of binding to DENV. The challenge with bead‐based virus detection is the need to concentrate the beads sufficiently to enable immunofluorescent detection of the virus. A U‐shaped electrode was used to concentrate the beads into a negative DEP region. An upstream V‐shaped electrode helped to guide the desired amount of beads into the U‐shaped electrode collection area. The device used an AC signal of 1 MHz frequency, and an additional drag force to move particles toward the capture electrode. This architecture required all forces to be balanced for successful capture. Whole viral particles were then flowed into the chamber and captured on the collected beads. These captured DENV where then detected by flowing a primary Anti‐DENV antibody, which bound to the captured DENV, followed by fluorescently conjugated donkey anti‐rabbit IgG secondary antibody that bound to the anti‐DENV antibodies.

### 3.3 Circulating extracellular vesicles

Tissues throughout the body, including those from diseased or damaged tissue, release a variety of different nanoparticles into circulation either through active or passive mechanisms. These nanoparticles carry different biomarkers on both their membranes and within their cytosol that relate back to the originating cells, including biomarkers related to different disease states such as cancer [51]. These nanoparticles are of clinical interest because the biomarkers they carry could be used to aid physicians in diagnosing various diseases, including early stage disease such as cancer. An important class of these particles includes extracellular vesicles (EVs) such as exosomes. Cellular organelles and organelle fragments present in circulation are also of interest for the biomarkers they potentially carry.

**Figure 5:**
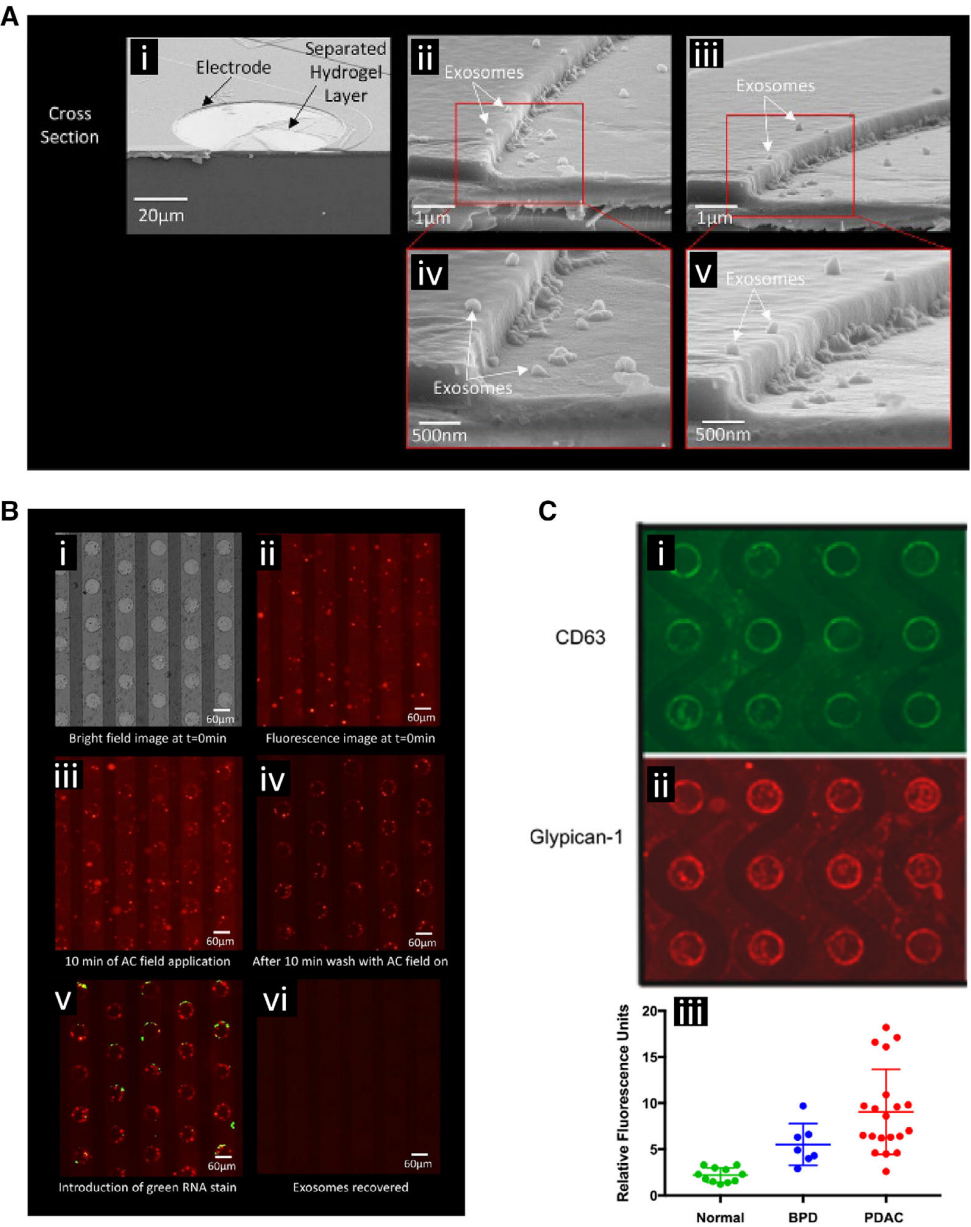
DEP‐based collection and detection of extracellular vesicles (EVs), including exosomes, and associated cancer biomarkers. **(A)** Scanning electron microscopy images showing exosome collection around the edges of circular electrodes of the DEP chip where the field strength was the highest. **(i)** Cell culture derived exosomes were collected from a buffer, the chip freeze dried, and then snapped in half to give cross‐sectional views of the collection. **(ii and iii)** Further magnification showing exosome collection at the electrode edge. **(** **iv and v)** Further magnification showing individual collected exosomes. Reprinted (adapted) with permission from [52]. Copyright 2017 American Chemical Society. **(** **B)** Collection of fluorescently labeled cell culture derived exosomes spiked into human plasma. **(i)** Bright field view of the electrode array. **(ii)** Fluorescent image showing distribution of EVs and exosomes before collection. **(iii)** Collection of EVs and exosomes around the electrode edges. **(iv)** The bulk plasma was washed away purifying the collected EVs and exosomes. **(v)** The collected particles were stained for biomarker content, in this case a green RNA stain was applied. **(vi)** The DEP field was reversed releasing the collected particles for subsequent off chip analysis. Reprinted with permission from [52]. Copyright 2017 American Chemical Society. **(C)** Detection of cancer‐associated biomarkers carried by the collected EVs and exosomes. The collected EVs and exosomes stained positively for the **(i)** CD65 biomarker and **(ii)** glypican 1 biomarker. **(iii)** Quantification of glypican 1 levels using DEP collection and immunostaining across different patients showed a significantly higher level of expression in the pancreatic ductal adenocarcinoma (PDAC) patients compared to normal controls. Benign pancreatic disease (BPD) also showed significantly elevated levels. Reprinted with permission from [53]. Copyright 2018 American Chemical Society.

Ibsen et al. demonstrated DEP collection of EVs, including exosomes, from undiluted human plasma (Fig. **5A** and **B**) [52]. Normal human plasma samples, spiked with concentrated exosomes from cell culture media, were run using DEP chips manufactured by Biological Dynamics. EVs were successfully isolated from the plasma with 10 min of DEP collection at 10  kHz followed by an *in situ* 10‐min wash with 0.5× PBS. Exosomes were analyzed using immunostaining techniques. Endogenous exosomes from a breast cancer patient were also collected from an undiluted plasma sample and successfully immunostained for the CD63 biomarker. Exosomes collected from buffer were analyzed on the chip using SEM. After collection, the chip was freeze dried to prepare for SEM imaging as well as to preserve the original morphology of the collected exosomes. EVs have been shown to be important biomarkers for many diseases.

Lewis et al. demonstrated DEP‐based rapid isolation of EVs directly from undiluted whole blood, plasma, and serum, and then used *in situ* immunostaining to quantify cancer biomarker expression using chips manufactured by Biological Dynamics (Fig. 5C) [53]. After DEP collection and a 0.5× PBS wash, directly conjugated antibodies against the biomarkers glypican‐1 and CD63 were flowed onto the chips without any additional processing steps. Glypican‐1 expression levels were shown to be significantly increased in pancreatic ductal adenocarcinoma (PDAC) and benign pancreatic disease (BPD) patients relative to healthy controls. Twenty PDAC patient samples were identified from 11 healthy patient samples with a 99% sensitivity and 82% specificity. CD63 expression levels in PDAC were not statistically significant between patients with BPD and normal groups nor PDAC and BPD groups. Also, ten colon cancer patient samples were analyzed and the three with metastatic diseases were identified.

Lewis et al. also demonstrated the ability of the DEP microarray platform and on‐chip biomarker immunostaining to detect the presence of brain tumors [54]. Glial fibrillary acidic protein (GFAP) and Tau were used as biomarkers to characterize meningioma, brain metastasis, and glioblastomas. Seventeen patients with tissue‐confirmed glioblastoma, brain metastasis, or meningioma were analyzed using the DEP chip system. These patients exhibited 65% higher average GFAP and 94% higher average Tau levels than the maximum observed in the control group. These biomarkers have also been reported to be expressed in patients who have experienced trauma, stroke, or Alzheimer's disease. These results support the use of plasma EVs for future work to diagnosis glioblastoma.

Sonnenberg et al. showed DEP capture of stained mitochondria from a 1.46 mS/cm storage buffer and of stained bacteriophages spiked into whole blood [55]. The mitochondrial protocol required prestaining of the mitochondria along with a significantly greater signal strength at 10 kHz for 30 min to achieve effective concentration and capture. Although collected from a buffer, this demonstrated that cellular organelles were capable of being collected using DEP.

A key aspect of these different studies has been the ability to perform the DEP collection of EVs in whole plasma and whole blood. Pulling these particles directly from undiluted biological samples is an important step towards clinical translation of DEP techniques for use with disease screening. By minimizing the manipulation of the biological fluid, the amount of required labor and processing time is reduced, both of which are important aspects for point‐of‐care devices and clinical diagnosis applications. In these studies, the ability to work in high conducting fluids was achieved through the application of a porous hydrogel layer over the surface of the electrodes developed by Biological Dynamics. The hydrogel prevented direct contact of the metal electrode with the bulk fluid, but still enabled the electric field to penetrate into the sample creating a DEP force on the EVs in the biological fluids. Expanding the ability of DEP to work directly in biological fluids is important for clinical applications. This also opens the door for future applications of DEP to early stage cancer detection where EV collection and cancer biomarker characterization could be widely used for cancer screening.

### 3.4 Circulating genetic material

Circulating DNA and RNA are another important class of materials released into the blood stream from tissues throughout the body and regions of localized disease where cells are damaged and are undergoing cellular lysis or through particle secretion processes [56]. The concentration levels of these nucleic acids in circulation is important as well as the mutations they carry that can indicate various disease states in the originating tissue, including mutations related to the development of cancerous tumors [57].

Jones et al. developed an iDEP microfluidic device that continuously separated DNA molecules by size [58]. The device contained insulating flow chambers with an inlet and five different outlets, where one outlet lay along the centerline of the device and the other four were directed radially outward from the junction. A 20 μm constriction following the 100 μm main flow channel generated electric field gradients, which imparted a DEP force of varying magnitude on different particles in the flow stream, causing the particles to deflect within the stream. DNA particles of different sizes were stratified into the different outlets with high molecular weight DNA (experiencing negative DEP) following the flow lines to the central outlet. Smaller, low molecular weight DNA (undergoing positive DEP) was deflected far enough to be diverted into the peripheral outlets. The sorting efficiency was discovered to increase linearly with applied AC voltage for all four DNA analytes. Additionally, maximum sorting efficiency occurred at lower frequencies for all four DNA lengths (plasmid DNA at 1.0, 10.2, and 19.5 kbp and λ‐DNA at 48.5 kbp). Next, 10.2 kbp and 48.5 kbp DNA were simultaneously processed through the device to test size sorting, resulting in longer DNA collected in the center and smaller DNA flowing into the outer outlets.

Zhang et al. developed an all‐in‐one nucleic acid isolation and analytic device, which consisted of an eight‐chamber, multilayered device to electrokinetically isolate and process DNA particles for gene analysis [59]. The device offered a central chamber capable of performing cell lysis, DNA binding, washing, elution, and then PCR in the same reaction chamber. The group demonstrated the device's capacity to perform DNA isolation and gene analysis using the TaqMan probe based droplet‐in‐oil PCR assays.

Sonnenberg et al. showed that cancer‐related circulating cell‐free DNA could be extracted from leukemia patient blood samples (undiluted plasma and whole blood) [60] using DEP devices described in Krishan et al. [61, 62]. The DNA was first isolated using a 10‐min application of a 10 kHz AC signal, then rinsed for 10 min in 0.5× PBS while still under DEP. All DNA samples were then eluted from the device and analyzed using PCR amplification and sequencing. Manouchehri et al. was able to demonstrate that cf‐DNA collected using DEP from chronic lymphocytic leukemia patient plasma contained cancer‐related point mutations that matched the point mutations found in the genomic DNA of the cancer cells themselves [57].

Ibsen et al. demonstrated that RNA contained within exosomes collected from glioblastoma cell lines could be analyzed using PCR techniques after recovery using DEP [52]. The cell culture derived exosomes were spiked into normal human plasma and recovered using DEP. RT‐PCR followed by endpoint PCR showed the presence of EGFRVIII mutations in the RNA, mutations known to be present in the cell lines themselves thereby indicating that the RNA mutations could be used as a biomarker for cancer detection. The exosomes collected on the DEP chip could be lysed open releasing the RNA content using either heat or various concentrations of Tween 20 surfactant.

The ability of DEP to recover DNA and detect disease‐related mutations is important for liquid biopsy applications. Typically, to get genetic information from tumors or other diseased tissues, a tissue biopsy is required that can be an invasive procedure with associated risks especially when dealing with deep delicate tissues such the pancreas [63]. Blood draws on the other hand are minimally invasive and can be done routinely for continued monitoring purposes. Applying DEP techniques to liquid biopsy applications is advantageous because the process can be designed to require minimal blood sample preparation, which could reduce associated cost and labor making this an application area that could advance DEP technology toward future clinical use.

### 3.5 Single‐celled yeast and bacteria

A large amount of DEP research has focused on the manipulation and isolation of various particles within microfluidic platforms. A significant emphasis has been placed on manipulating single‐celled organisms. First achieved by Pohl and Hawk in 1966, the separation of live and dead yeast cells set the stage for thousands of biological experiments and hundreds of device designs [2, 4]. The separation and recovery of yeast and bacteria are of great interest in multiple applications including disease detection.

Zhao et al. developed an alternating current (AC) DEP microfluidic device for measuring lateral migration of yeast cells [64]. The device contained two metal electrodes separated from the main flow channel, which allowed the applied DEP force to selectively differentiate cells. Dielectrophoretic forces oriented the cells along the sides of the insulated primary flow chamber where they flowed through respective outlets. The outlets were specifically fabricated on both insulating layers to have widths that varied by 50 μm and the asymmetry of these features produced an electric field gradient that spanned the width of the primary flow chamber. Therefore, lateral migration was controlled by selecting a frequency that separated cell populations based on different crossover frequencies. As both live and dead yeast cells flowed along the center line of the primary chamber, a signal between the crossover frequencies was applied causing live yeast cells to move laterally toward the smaller outlet (high field gradient) while the dead yeast cells simultaneously migrated toward the larger outlet. This device shares a similar fundamental architecture to the original work by Pohl and Hawk, yet this unique device architecture allowed for a one‐step isolation and collection [4].

Gencoglu et al. demonstrated an iDEP technique using an insulating‐post architecture to separate yeast cells (6.3 μm average diameter) from 1 and 2 μm polystyrene beads [65]. They used a tapered AC signal to collect all three particles and concentrate them at the insulating post array. The AC signal gradually reduced in intensity while simultaneously introducing a DC offset over the course of the experiment. Once the signal became more asymmetrical, particles were selectively released starting with the smallest particles. The larger yeast cells were released once the signal became cyclical. The DEP force experienced by the different types of trapped particles was reduced at different rates depending on the properties of the particles resulting in their sequential release.

Koklu et al. demonstrated low conductivity DEP enhanced manipulation of *Saccharomyces cerevisiae* yeast cells using fractal gold nano‐electrodes. They combined their optimized, rough electrodes with a box‐shaped, castellated electrode configuration and used a low‐intensity 5 MHz signal to minimize electrothermal effects [66].

Moving from yeast to bacteria, Chen et al. fabricated a microfluidic DEP platform that was capable of trapping, counting, and detecting *Shewanella oneidensis* [67]. This device achieved rapid, label‐free detection of *S. oneidensis* using a castellated, or interdigitated, electrode configuration with a central flow channel. Bacteria were trapped by positive DEP and observed with real‐time fluorescence imaging and Raman spectroscopy.

**Figure 6:**
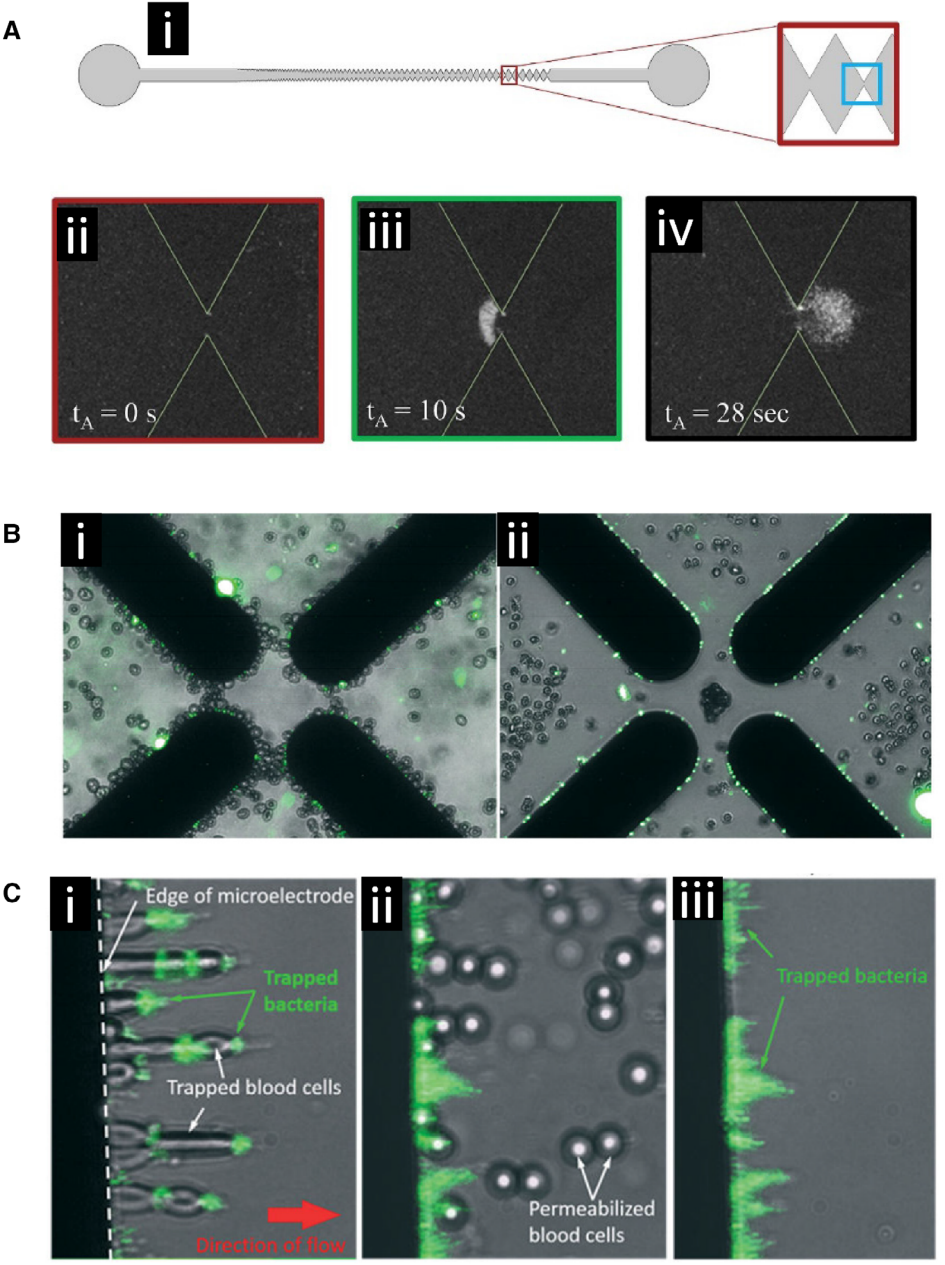
DEP‐based collection of bacteria. **(A)** Collection of *Listeria monocytogenes* 1/2a bacteria using a gradient‐iDEP microdevice. **(i)** Schematic representation of the channel geometry. **(ii)** Fluorescent image of the channel before collection. **(iii)** Bacteria are continuously trapped at the inlet side of the gate by negative DEP while the potential is applied. **(iv)** After the potential is removed, the collected bacteria are released. Reprinted from [68]. Copyright 2019, with permission from Elsevier. **(B)** DEP‐based separation of *Escherichia coli* from human blood cells. **(i)** Both the *E. coli* and the human erythrocytes were collected in positive dielectrophoresis (pDEP) at the edges of a quadrapole electrode array. **(ii)** Adding the ionophore monensin facilitated the transport of ions across cell membranes changing the crossover frequency of the erythrocytes causing them to undergo negative DEP (nDEP) under the same conditions and separated them from the intact bacteria, which were still collected in pDEP at the electrode edges. Used with permission from the Royal Society of Chemistry, from [73] Copyright 2017; permission conveyed through Copyright Clearance Center, Inc. **(C)** The separation of human blood cells from *E. coli* bacteria can be achieved using a flow system and a linear electrode array. **(i)** Blood cells and bacteria are both collected at the electrode edge. **(ii)** Introduction of saponin permeabilized the blood cells changing their crossover frequency causing them to be released from the electrodes. **(iii)** The bacteria redistributed after the loss of the blood cells but remained collected at the electrode edge. Used with permission from the Royal Society of Chemistry, from [73] Copyright 2017; permission conveyed through Copyright Clearance Center, Inc.

Crowther et al. demonstrated rapid collection and characterization of the important foodborne bacterial pathogen *Listeria monocytogenes* using a gradient‐iDEP microdevice (Fig. **6A**) [68]. They gathered epidemiological information by measuring the electrokinetic and dielectrophoretic properties of the three *L. monocytogenes* serovars 1/2a, 1/2b, and 4b enabling their differentiation from one another. Specifically, the electrokinetic velocity and dielectrophoretic trapping properties of the bacteria were measured, in conjunction with multiphysics modeling. The gradient‐iDEP microdevice used a sawtooth channel geometry where the distance between triangular insulator tips decreased down the length of the channel. The biophysical properties of the bacteria cells determined where along the channel they were trapped based on where the electrokinetic force balanced the dielectrophoretic force. This type of chip geometry was also shown to be able to differentiate three serotypes of *Escherichia coli* bacteria [69]. The startup company Cbio.io has licensed some of this technology.

Su et al. used a DEP device with a quadrupole electrode array to test the susceptibility of Gram‐negative bacteria to β‐lactam antibiotics [70]. This DEP antimicrobial assay was tested using four standard strains and 78 clinical isolates, spanning 13 important Gram‐negative bacteria species. The minimal inhibitory concentrations (MICs) of cefazolin, ceftazidime, cefepime, and doripenem were determined using both their DEP assay and standard broth dilution testing. Morphological changes, including cell elongation, cell swelling, or lysis, were directly observed within 90 minutes using susceptible bacteria under 1× MIC. The DEP and conventional assays were in 95.6% agreement with a major error rate of 2.9% for the DEP assay.

Koklu et al. used a square box, castellated microelectrode configuration to achieve negative DEP capture of bacterial spores from *Bacillus subtilis* and *Clostridium sporongenes*, and demonstrated device use under biologically viable conductivities [71]. The latter enabled research to be conducted using undiluted patient samples, rather than needing to mitigate electrochemical damage through dilution of the sample. They also showed the collection of bacteria from actual food matrices demonstrating that this technique could have applications to test the food supply for bacterial contamination. In the same publication, the group demonstrated global electroosmotic fluid flow across the device interface.

Park et al. used a high conductivity device, implementing a new multichamber microfluidic design with sequential flow and an angled linear gold electrode arrangement [72]. The device used positive DEP to separate target cells from biological fluids to selectively draw *E. coli* to a collection chamber allowing the remaining sample to flow through into a waste chamber.

D'Amico et al. constructed a DEP device using a quadrapole electrode configuration to isolate *E. coli* from dilute, low conductivity, red blood cell/bacteria cultures (Fig. 6B and C) [73]. The membrane‐less microfluidic DEP dialysis system successfully isolated and concentrated *E. coli* and *Staphylococcus aureus* bacteria using a label‐free capture and wash assay. They cultured *E. coli/S. aureus* in human whole blood, selectively isolated red blood cells and bacteria, and then re‐suspended red blood cells and bacteria into a dilution buffer solution. A permeabilization agent was added to the suspension and sample conductivity was then adjusted. The device leveraged the change in selectivity on the red blood cells after permeabilization, leaving *E. coli* and *S. aureus* unaffected. Researchers applied a 1 MHz AC signal to the device with a 10 μL/min flow rate. Under these conditions, the system successfully isolated 79 ± 3% of the targeted bacteria and they did not find a drop in device efficiency for samples under $10^{5}$ cells per process cycle. Quantitative PCR was performed to show a 307‐fold enrichment of bacterial DNA relative to the human DNA content in the original sample.

De Ninno et al. developed a microfluidic impedance cytometry device consisting of five coplanar electrodes for single cell analysis, including particle sizing, without the need for flow focusing [74]. The design of the chip created a specialized signal shape that was used to account for particle trajectory through the creation of a new metric called relative prominence. The relative prominence encoded for the particle's trajectory height as it flowed over the electrodes and was used to correct for the estimated particle sizes. They demonstrated this technique on populations of beads and then on *S. cerevisiae* yeast showing that two different populations of yeast cells were present in the sample. In addition to sizing, the device could be used for cell counting as well.

Adekanmbi et al. showed that the absorption of rare earth elements by Cupriavidus necator bacteria changed their DEP crossover frequency [75. These elements included neodymium Nd^3+^, samarium Sm^3+^, and europium Eu^3+^. The degree of change was related to the amount of absorption which was directly proportional to the metal dosage and incubation time. This enabled the use of the bacteria as a biosorbent and dielectrophoresis as the quantification technique for the rare earth elements. This demonstrated the ability of DEP to detect biosorbtion processes with potential applications to a wide range of different analytes of interest.

These different chip designs and implementations, all of which successfully manipulate, collect, and characterize single‐celled organisms, highlight how well suited DEP is to these types of applications. The wide range of different species that DEP has successfully been able to work with indicates that there is plenty of space for future expansion into important applications. Microbes play such an integral role in the environment, food safety, and in health care that developing high‐speed separation, detection, and characterization techniques, such as what DEP can achieve, will fill important current and future needs within each field.

### 3.6 Mammalian cell sorting and manipulation

Separation and collection of mammalian cells has been a major focus of DEP research and development. Determining the crossover frequency of cells within a population can be used to determine different properties about the cells themselves, how they respond to different environmental conditions, and separate and recover desired cell populations from mixtures of different cell types. This has important applications in basic cellular biology studies, disease detection, and diagnosis.

#### 3.6.1 Cancer cell manipulation

DEP offers some unique applications for cancer detection, diagnosis, and treatment. Researchers have shown DEP‐based devices selectively isolating target cancer cells based on their type and characteristics. This is important for developing techniques that use DEP for early‐stage cancer detection and also for differentiating cancer cells based on levels of aggressiveness which could inform therapeutic decisions.

Henslee et al. developed a contactless DEP (cDEP) device, using fluid electrode channels, to isolate breast cancer cells from a heterogeneous mixture of live cells [76]. Their study included MCF10A, MCF7, and MDA‐MB‐231 human breast cells, which represented early, intermediate, and late‐staged breast cancer, respectively, and each cell type exhibited distinct responses to the trapping signal. MDA‐MB‐231 cells were selectively isolated from a heterogeneous mixture of all three cell types and the frequency range over which this was possible was found to vary depending on the applied voltage.

Douglas et al. developed a microfluidic chip that coupled fluid flow with orthogonal DEP forces to displace and enrich cancer cell subpopulations expressing an aggressive phenotype [77]. cDEP was used to attract the cells with aggressive phenotypes to posts in the chamber. Two subtypes of the mouse ovarian surface epithelial (MOSE) cell line were used to illustrate the device's ability to separate cells having genotypic similarities but varying levels of malignancy. The MOSE‐LTICv subtype represented malignant cells and the MOSE‐L subtype represented slow developing disease. They were able to show that separating cells with similar bioelectric properties could be improved by optimizing the DEP/drag force balance via adjusting the flow rate. Since these cell line subtypes originate from the same original cell line, they can be used to model the colonial expansion of *in vivo* tumor evolution.

Soltanian‐Zadeh et al. developed an iDEP device to investigate the response of cancer cells to chemotherapy drugs [78]. Their system selectively trapped the breast cancer cell lines LCC1 and LCC9 and analyzed the effect that Obatoclax, an anti‐cancer drug, had on these cells. Aliquots of cells were exposed to 100 nM, 500 nM, and 1 μM Obatoclax, with each increased concentration of drug exposure resulting in a higher crossover frequency for both cell lines. Untreated LCC1 had a crossover frequency of 700 kHz, which increased to 900 kHz after introduction of 1 μM Obatoclax and LCC9's initial crossover frequency shifted from 100 to 400 kHz with 1 μM Obatoclax. Trapping efficiency was altered by Obatoclax as well: at 900 kHz, LCC1 cells had an increase in trapping efficiency with 1 μM Obatoclax treatment. At 1 MHz, the drug resulted in a decrease in trapping efficiency. For LCC9, trapping efficiency between 100 and 800 kHz went down with an increase in drug concentration, but efficiency increased between 900 kHz and 1 MHz.

Mahabadi et al. demonstrated whole cancer cell phenotyping by using DEP to evaluate chemotherapy drug response [79]. Using the 3DEP instrument from DEPtech, cancer cell lines were analyzed for changes in their membrane capacitance and cytoplasm conductivity after having been exposed to anticancer therapy. Observed changes to the human head/neck squamous cell carcinoma cell line HN5 after exposure to the therapeutic Iressa included a decrease in cytoplasm conductivity, membrane capacitance, and membrane conductance and an increase in cytoplasm permittivity. These changes could be detected within 6 h of initial exposure. The T47D cell line was not susceptible to these treatments and showed no change in properties. The technique was able to assess therapeutic benefits from combination therapies as well. The technique offers a quick and cost‐effective method to determine the effectiveness of different treatments. Future applications include evaluation of different treatments on cells derived from cancer biopsies, which in the future could help select effective treatments for patients.

Mansoorifar et al. constructed a series of cell capture and characterization devices utilizing a SU‐8 epoxy microwell array (30 μm microwell diameter) with their well‐developed gold electrode impedance platform [80, 81]. The group used their dielectric spectroscopy method to optimize frequency ranges to achieve the most effective DEP forces. The device was used over an 8‐h window with low conductivity buffers to determine cell viability of PC‐3 prostate cancer cells exposed to 100 μM Enzalutaminde. The performance of their chip designs offer the possibility of a system that can quantify drug efficacy in higher conductance samples.

**Figure 7:**
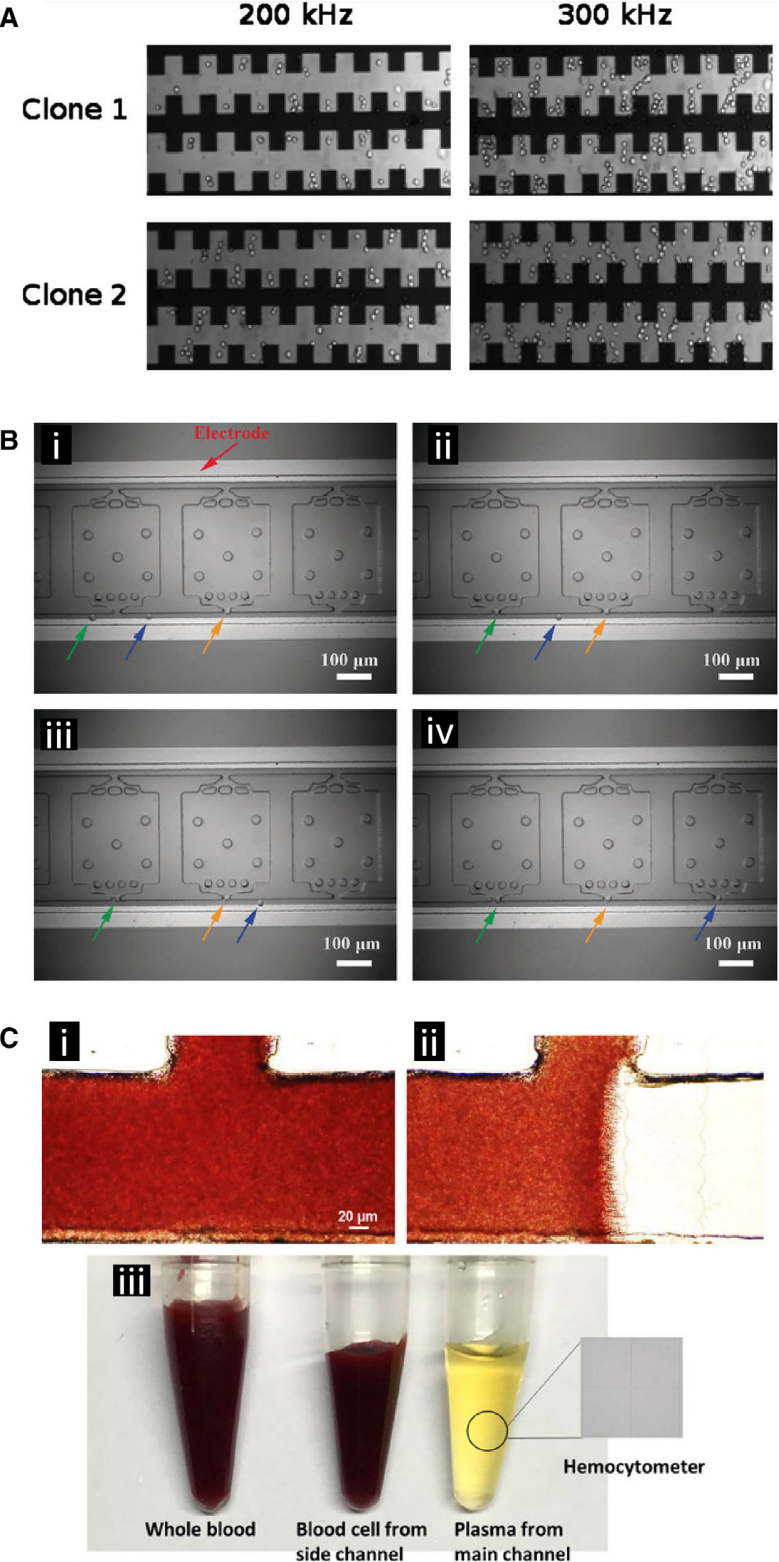
DEP‐based spatial manipulation of mammalian cells. **(A)** DEP separation of two clones of B16F10 mouse melanoma cancer cells from the same origin. Using a castellated electrode array, at 300 kHz, clone 1 exhibited negative DEP (nDEP) and clone 2 exhibited positive dielectrophoresis (pDEP). The main measurable difference between the two clones was that clone 1 had significantly higher levels of melanin content than clone 2. Used with permission from the American Institute of Physics, from [82] Copyright 2010; permission conveyed through Copyright Clearance Center, Inc. **(B)** DEP‐based manipulation and localization of single cancer cells. **(i–iv)** Single chronic myelogenous leukemia K562 cells (highlighted by the arrows) were manipulated into position at the entrance to an analysis chamber using iDEP. The narrow entrances to the chambers created local electric field maxima trapping individual cells in pDEP as they flowed past. The cells were subsequently released from the chamber entrance, sealed in the chamber, and then analyzed for genetic expression using LAMP amplification. Adapted with permission from [84]. Copyright 2018, WILEY‐VCH. **(C)** Use of a linear electrode array to separate plasma from red blood cells. **(i)** The main channel with the side shunt channel at the top before application of the AC field. **(ii)** During AC field application the electrode array creates an nDEP force on the red blood cells forcing them into the side shunt channel and allowing plasma to continue forward. **(iii)** The resulting purified plasma collected from the main channel as well as the concentrated red blood cells collected through the side channel. Adapted with permission from [93]. Copyright 2018, WILEY‐VCH.

Sabuncu et al. used a castellated electrode design to selectively separate and capture B16F10 clonal mouse cells based on melanin concentrations (Fig. **7A**) [82]. In the same study, an impedance spectroscopy device, using a 4‐electrode chamber design, was used to interrogate T‐cell leukemia Jurkat cells.

Koklu et al. used a rough, or granulated, electrode to increase the sensitivity of their low‐frequency impedance measurements [83]. They further reported a greater biocompatibility between granulated gold nano‐electrodes and the leukemic Jurkat T cells as compared to platinum black electrodes, which also simplified device construction.

Qin et al. demonstrated a self‐digitization dielectrophoretic (SD‐DEP) chip design capable of trapping single cells within individual wells enabling single cell nucleic acid analysis (Fig. 7B) [84]. They used an iDEP technique to trap individual K562 cancer cells at the entrance of the wells allowing non‐trapped cells to pass though the flow channel. The cancer cells were then moved into their individual wells where the cells were then lysed and analyzed. Using DEP to manipulate individual cells and confining them to wells in this manner minimized contamination, decreased the dilution of the cell lysate, and kept the DNA and resulting signal concentrated thereby increasing the sensitivity of the overall assay. This technique could enable single‐cell research for precision medicine applications.

Keim et al. developed a label‐free DEP‐based method to conduct analysis on the biophysical properties of single cells [85]. Tri‐dimensional pillar electrodes were used to create separate micro‐cages to trap individual cells and induce a rotation of the cell. An electrorotation spectrum for each cell was created by measuring the rotational speed of the cell as a function of applied electric field frequency enabling the study of the cell's dielectric properties. Multiple cells could be multiplexed on the same chip in different traps. The system was validated using known cell types and was able to determine the membrane capacitance for M17 neuroblastoma cells (7.49 ± 0.39 mF/m^2^).

Thomas et al. demonstrated a new microfluidic cell sorting DEP device that used negative DEP to create a “dielectrophoretic virtual channel” along the middle of the actual flow channel [86]. This virtual channel could be reconfigured by switching the polarity of the electrodes to direct incoming cells along different paths and into different collection outlets. The automated system used optical imaging to make decisions about which way to send incoming particles based on fluorescent labeling and the characteristics of the particles. This allowed for rejection of debris. The system was capable of isolating high purity populations of cells from a mixture including osteosarcoma and human bone marrow cells.

#### 3.6.2 Blood cell separations

Blood is a complex mixture of particles including many different cell types and circulating nanoparticles. The minimally invasive nature of blood draws makes blood an easily accessible and important source of cells for clinical diagnosis and basic research. Separating these blood cells from particles within the plasma and separating cell types from one another can be a challenge especially when dealing with different types of white blood cells that have similar sizes and densities. There can be a more significant contrast in the dielectric properties between these particles compared to their physical properties, which allows DEP to create differential forces upon them.

Elitas et al. developed an electrochemically stable microfluidic chip with 3‐D carbon electrodes, using a multistep photolithography process on a silicon wafer, for use with cell characterization [28, 87, 88, 89]. The electrodes were 100 μm tall and 50 μm in diameter with 15 μm spacing in between. U937 cultured cells were suspended in a sucrose/glucose/BSA‐low‐conductivity buffer. The respective crossover frequencies of the U937 monocytes and U937 monocyte‐differentiated macrophages were found to be 17 kHz and 30 kHz. They then characterized the monocyte/macrophage cell suspension under no‐flow conditions using a series of tests across a range of frequencies from 1 kHz to 20 MHz. DEP cell separation was then demonstrated between the specific subpopulations in a mixed sample, yielding a 70% enrichment, as confirmed by fluorescently activated cell sorting analysis.

Sonnenberg et al. demonstrated simultaneous manipulation of nanoparticles and blood cells under high conductance conditions [90]. They were able to achieve clear negative DEP migration of red and white blood cells in between electrodes of their array and positive DEP collection of nucleic acid and protein nanoparticles on the periphery of the electrode edges. They used diluted human blood samples with a high conductance buffer and applied a 10 kHz signal.

#### 3.6.3 Bulk red blood cell separation from plasma

Given the prevalence of DEP microfluidic platforms for cell sorting, it is conceivable that DEP could be used for continuous, efficient, and high‐throughput blood plasma isolation. High conductance limitations of DEP remain a challenge due to dielectric loss factors at the typical frequencies used to manipulate cells in blood or in near‐biological buffers (≈1.4 S/m). A potential solution is to reduce the conductivity of the media by exchanging cells into a lower conductance buffer. This presents several challenges by introducing a number of extra steps, and places substantial osmotic pressure on suspended cells that can reduce their viability. Other DEP blood processing devices that operate at normal biologic conductivities are more limited by throughput capacity. This lower throughput capacity may not be a factor when dealing with rare samples that have a small available plasma volume, such as from a drop of blood.

Yan et al. constructed a microfluidic chip using a series of layered flow channels that allowed for selective separation of red and white blood cells, leaving the main flow chamber with clean plasma. The blood was diluted before DEP separation to control sample conductance [91, 92].

Yang et al. fabricated a DEP chip for continuous extraction of blood plasma from undiluted human whole blood, requiring no pre‐processing steps and achieving nearly 100% plasma purity and a plasma yield of 31% (Fig. 7C) [93]. The device was composed of a main channel with an additional, perpendicular side channel at its midpoint. Indium tin oxide (ITO) electrodes were placed adjacent to the side channel and repelled red blood cells from continuing down the main channel using negative DEP. Hydrodynamic forces then pushed the red blood cells into the side channel while blood plasma flowed unimpeded past the electrodes to the end of the main channel. Pure plasma was extracted from a drop of whole blood, approximately 7 μL, in less than 15 min. Reducing the distance between the electrodes or fabricating electrodes with finer edge features could produce a greater electric field gradient with a lower operating voltage. More stable metal electrodes could replace the ITO to allow for greater processing speed, but this device offered a way forward for future efficient on‐chip blood plasma extraction. The external force and injection flow rate in this device could be optimized to meet the efficiency and throughput needs dictated by commercial and clinical applications of blood plasma extraction.

#### 3.6.4 Circulating tumor cells

The cells shed by tumors into circulation are of great interest because of the information they carry about the tumor itself and their metastatic potential. These circulating tumor cells (CTCs) are present in very low concentrations, which makes recovering them from the greatly more concentrated red and white blood cells also in circulation a challenge. Often these CTCs have differences in their dielectric properties compared to the other cells that enable DEP‐based separation.

Moon et al. developed a continuous flow system that used DEP to separate CTCs from other blood cells [94]. Their design used two separation techniques in the same chip. The first was a hydrodynamic separation technique to remove a large amount of the red blood cells followed by DEP to enhance the separation of the remaining red blood cells from the desired CTCs. The DEP forces acted differently upon the two cell types and due to differences in their dielectric properties forced most of the red blood cells to flow into a different exit channel. They used human blood samples spiked with human breast cancer cells (MCF‐7) for these experiments and observed a 162‐fold increase in cancer cell concentration with removal of 99.24% and 94.23% of the red and white blood cells, respectively.

Boyer et al. was able to use a different CTC collection strategy to characterize Merkel cell carcinoma cells collected from patient blood samples [95]. They used the RosetteSep‐DEPArray workflow from Menarini‐Silicon Biosystems. This two‐stage process first enriched the CTCs in the patient blood sample by an immunoprecipitation‐based negative selection process to remove unwanted cells. The CTCs were enriched through this process and then sorted according to their phenotype and staining pattern using the DEPArray system that combined microelectronic and microfluidic processing across a 300 000 micro well tray for ultra‐high precision cell sorting and selection. Each DEP “cage” had the capacity to hold and manipulate a single cell by coordinating signals between the adjacent cages [96, 97, 98]. This allowed for cell selection and characterization using a series of immunostains for different biomarkers. Cells with the characteristics of the desired CTC were then selected for further DNA analysis. Using this process, CTCs were found in 42% of patient samples.

### 3.7 Cell transfection

An important application in cell biology research and in industrial processes is the transfection of cells with DNA plasmids in order to have the cells express the proteins encoded by the DNA. This changes their behavior in various model systems and produces proteins for industrial applications. Electroporation is a common method by which to do this, but requires the cells to be positioned near the electroporation electrodes.

A unique application formulated by Chang et al. allows for improved bacterial transfection [99]. They were able to develop a versatile loading technique with gene editing, drug delivery, and molecule loading potential using a silicon, 3D nanochannel electroporation platform. This 3D nanochannel electroporation (NEP) device can precisely and benignly process 60 000 cells/cm^2^ via a two‐step transfection protocol. Positive DEP selectively positions and holds single cells in silicon nanochannels for optimized subsequent electroporation. The difficult‐to‐transfect cell line NK‐92 was tested on the 3D NEP‐DEP device, resulting in placement of 79% of the cells in nanochannels and, of those cells, 93% were successfully transfected.

### 3.8 Stem cells and cancer stem cells

Stem cells are of particular interest because they have the potential to differentiate into various cells types, which makes characterizing them essential for the study of basic tissue development, embryology, and various medical applications including regenerative medicine and tissue engineering. The intercellular signaling that occurs with the structural patterning of the stem cells, including their proximity to one another, along with external signaling cues from different chemicals and growth factors, can influence their differentiation pathways. Recovering stems cells from heterogeneous populations with minimal disturbance enables the study of their properties with minimal confounding stimulation. Cancer cells that have stem cell like properties may also play an important role in cancer progression.

Yale et al. demonstrated that the whole‐cell membrane capacitance of neural stem and progenitor cells reflects their differentiation potential [100]. They showed that cell surface carbohydrates contributed to both the observed membrane capacitance differences and influenced cellular differentiation fate. The presence of highly branched N‐glycans on the cell surface lead to an increase in the membrane capacitance and also resulted in a higher probability of differentiating into astrocytes instead of neurons. DEP was used to sort the stem cells. Cells biased to differentiate into astrocytes were attracted to the DEP electrode edges allowing the neuron‐biased cells to be washed away thereby sorting for the astrocyte‐biased cell population. The short time frame of the DEP sorting was shown to not alter the cells' differentiation, survival, or proliferation [101].

**Figure 8:**
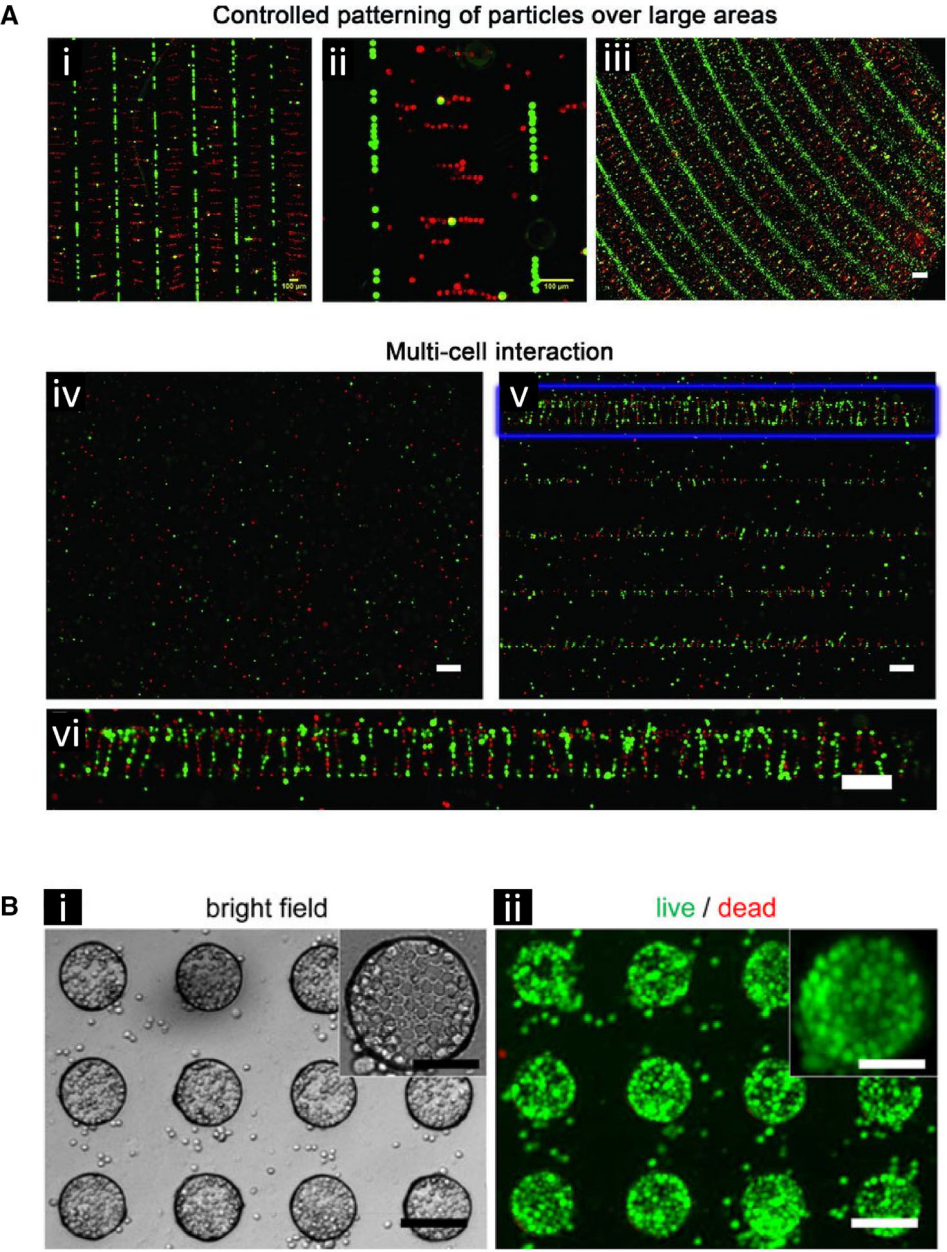
DEP‐based spatial patterning of stem cells. **(A)** Patterning of stem cells and polystyrene beads using differently shaped electrode arrays with subsequent encapsulation through gelation of the surrounding media. **(i)** Linear patterning of C2C12 cells in red (positive dielectrophoresis [pDEP]) and polystyrene beads in green (negative DEP [nDEP]). They were subsequently encapsulated in a 15% PEGDA 700 gel. Scale bar = 100 μm. **(ii)** Zoomed in image of panel (i). Scale bar = 100 μm.**(iii)** Spiral electrodes were used to make curved patterns of mESCs cells in red (pDEP) and polystyrene beads (green) (nDEP) with subsequent encapsulation in a 20% PEGDA 700 gel. Scale bar = 200 μm. **(iv)** Without DEP, mESCs cells dyed red and green have a homogenous distribution. **(v)** With DEP, the mESCs cells from panel (iv) formed linear patterns with subsequent encapsulation in a 15% PEGDA 3400 gel. **(vi)** A zoomed in image of panel (v) showing cell/cell interactions. Adapted with permission from [102]. Copyright 2012, WILEY‐VCH. **(B)** Mouse embryonic stem cells patterned into curricular microwells using DEP. **(i)** The bright field image of the patterned stem cells. Scale bars: 100 μm, inset 50 μm. **(ii)** The live dead stain analysis after 1 day where green shows the live cells. Scale bars: 100 μm, inset 50 μm. Reprinted by permission from Springer Nature: [103]. Copyright 2010.

Bajaj et al. developed custom microelectrodes for DEP patterning of mES and C2C12 skeletal muscle myoblasts (Fig. 8A) [102]. The DEP platform, running between 1 and 10 MHz, was coupled to a stereolithography apparatus to both pattern and encapsulate mESs, mouse embryoid bodies, and C2C12 spheroids in hydrogels with physiologically relevant stiffness. Large 10 mm × 10 mm arrays of hydrogels were created, illustrating the ability to fabricate three‐dimensional microstructured cellular patterns. Cells exhibited a pearl chain structure after DEP pattering, which allowed cell–cell contacts to be established and potential fate decisions to be investigated. Encapsulated mESs showed a viability of 94% and 91% when patterned with conventional stereolithography and DEP‐enhanced stereolithography, respectively. Next, mouse embryoid bodies and C2C12 spheroids were formed before positive‐DEP patterning and hydrogel encapsulation. Live/dead assays evaluated after 1, 3, and 5 days showed the viability of the mouse embryoid bodies in the hydrogel, and demonstrated spatial organization of both mESs and cellular spheroids in the hybrid DEP‐stereolithography platform. High‐throughput investigation of multicell and multimaterial interactions that mimic the cells' *in vivo* microenvironment could be achieved using this technology.

Tsutsui et al. developed a microfluidic iDEP‐based device that used an external flow to structurally pattern mouse embryonic stem cells (mES) in PEG microwells (Fig. 8B) [103]. The microstructured hydrogel was patterned on a planar ITO electrode and formed positive DEP traps for the cells. The device achieved a seeding density of 10^7^ cells/mL and successful cell trapping occurred in less than 1 min. Using live/dead fluorescence assays, it was confirmed that the isolated cells formed viable and homogeneous monolayers within the microwells, showing potential for future on‐chip drug testing, complex tissue microenvironment testing, etc.

Song et al. fabricated a continuous‐flow DEP device that sorted stem cells from their differentiated products [104]. Interdigitated metal electrodes were positioned at 45° to the flow trajectory to selectively steer particles within the stream. Osteoblasts were deflected laterally and followed a zig‐zag pattern due to the combination of DEP and fluid forces, whereas human mesenchymal stem cells remained on their original trajectory due to weak DEP forces. Human mesenchymal stem cells (hMSCs) were collected with a maximum efficiency and purity of 92% and 84%, respectively, while deflected osteoblasts were collected with up to a 67% efficiency and 87% purity. The partial differentiation of hMSCs may describe the heterogeneous sorting achieved by this device.

Ahadian et al. used interdigitated platinum electrodes fabricated on a glass substrate to align graphene within PEG hydrogels to affect cellular behavior and ultimately produce 3D stem cell scaffolds [105]. Exfoliated multilayer graphene, at concentrations of 2.55 mg/mL and 5.1 mg/mL, was treated with bovine serum albumin and unpolymerized PEG. The treated graphene was tested for multiple electrical and mechanical properties. Horizontal alignment occurred with a 1 MHz signal across the platinum electrodes, and an additional ITO electrode oriented above the device served to align the graphene vertically. The suspension was then stabilized by cross‐linking the PEG with UV light. The aligned graphene was shown to increase the electrical conductivity and the Young's modulus of the hybrid graphene‐PEG hydrogels. Each hydrogel was introduced to a serum‐free media containing undifferentiated mouse stem cells, and then tested as a scaffold in a 37°C, 50% CO_2_ incubation environment. After 5 days of monitored incubation, the horizontally aligned graphene showed an increase in the percentage of viable cells, whereas randomly aligned and pristine hydrogels showed minimal increases.

Alinezhadbalalami et al. fabricated a cDEP device with an array of insulating posts, which was used to isolate prostate tumor cells with stem‐like properties from other malignancies, and separate aggressive ovarian cancer cells from early stage cells [106]. Additionally, cells from the U251 glioblastoma cell line and its spheroid counterpart, SF‐U251, were differentiated based on their respective dielectric properties. The SF‐U251 cells required higher voltages for successful trapping than the U251 cells and also exhibited greater variability in the trapping voltage. Direct immunofluorescence of samples was also demonstrated on the devices. These two glioblastoma subpopulations expressed a distinct difference in nestin and GFAP transmembrane expression, respectively, which may have led to the distinct DEP characteristics. This demonstrated the ability of DEP to separate cells that had been grown under different culture conditions.

As with single‐celled organisms, the diverse set of DEP chip designs and approaches described in this section demonstrates how well‐suited DEP techniques are to detecting and studying single cells derived from mammals. It can be applied to cells derived from culture lines and to cells derived directly from patients making it a versatile tool to study both the basic biology behind disease and for detection of the disease itself. Both areas benefit from the ability to isolate and characterize cells of interest from heterogeneous populations. The wide range of different cell types DEP can be applied to, including blood cells, cancer cells, and stems cells, demonstrates the potential the technique has for current and future use in research and clinical applications and gives rationale for further expansion and development of the technique to study other cell and disease types.

Using DEP to help analyze and characterize fixed cells or cells derived from tissue biopsies would allow access to large existing biobanks of samples and could add beneficial characterization to existing analysis techniques for these valuable resources. Future areas that are important to develop for greater clinical applicability of DEP techniques include the ability to run DEP in high conducting fluids that more closely approximate cell culture media and biological fluids and continuing the development of automated systems to run these separations and analyses.

### 3.9 Electrode development for biological fluid applications

To operate in the high conductance and complex mixtures that are physiological fluids, the electrodes of DEP devices need to be specially designed. Heineck et al. explored the limits of DEP device function using two different architectures, with the end point being either electrode failure or sample degradation [22]. Sinusoidal signals were tested across different frequencies and voltages. It was found that biological fluids and buffers, naturally corrosive and highly conductive, were able to electrochemically degrade platinum at low frequencies and cause the medium to locally hydrolyze and/or boil at high frequencies, with a transition frequency around 10 kHz. This study pointed to favorable frequency windows where targeted particle capture could be maximized while mitigating degradation of both the electrode and the target material.

## 4 Synthetic Nanoparticles

Over the last 10 years, in addition to the application of DEP to biologically derived particles, there has been exciting developments in DEP applications using and manipulating synthetic particles. Nanoparticles have always been a challenge to physically manipulate, orient, arrange, and sort due to their incredibly small sizes and often large numbers. Here, DEP has a unique ability to create forces that preferentially act upon nanoparticles within certain size ranges and material composition. The following highlights some of the applications that DEP has uniquely contributed to with synthetic nanoparticles.

### 4.1 Nanobeads

Spherical nanoparticles, or nanobeads, comprised of various materials can be made with tight size distributions and with various surface functionalization and physical characteristics. This makes them ideal to work with when developing new DEP techniques, demonstrating proof of concepts, and demonstrating the fine discrimination abilities of new technology.

Polniak et al. leveraged the electrical properties of different particles to separate particle populations by carefully manipulating the respective dielectrophoretic, electroosmotic, and electrophoretic forces via the electrical signal and DC bias [107]. This allowed them to separate a mixture of extremely similar 10 μm particle populations, with either green or red fluorescent functionalization.

Perez‐Gonzalez et al. looked at different iDEP‐based chip designs in order to reduce DC voltage requirements needed to trap particles [108]. They found through COMSOL Multiphysics simulations and experimentation that reducing the number of sequential insulating posts longitudinally distributed across the channel reduced the required voltage necessary to trap particles within the array. This is important because lower voltages will result in less heating of the sample. They found that a single column of posts was most effective at capturing both 200 nm and 1 μm fluorescent particles.

**Figure 9:**
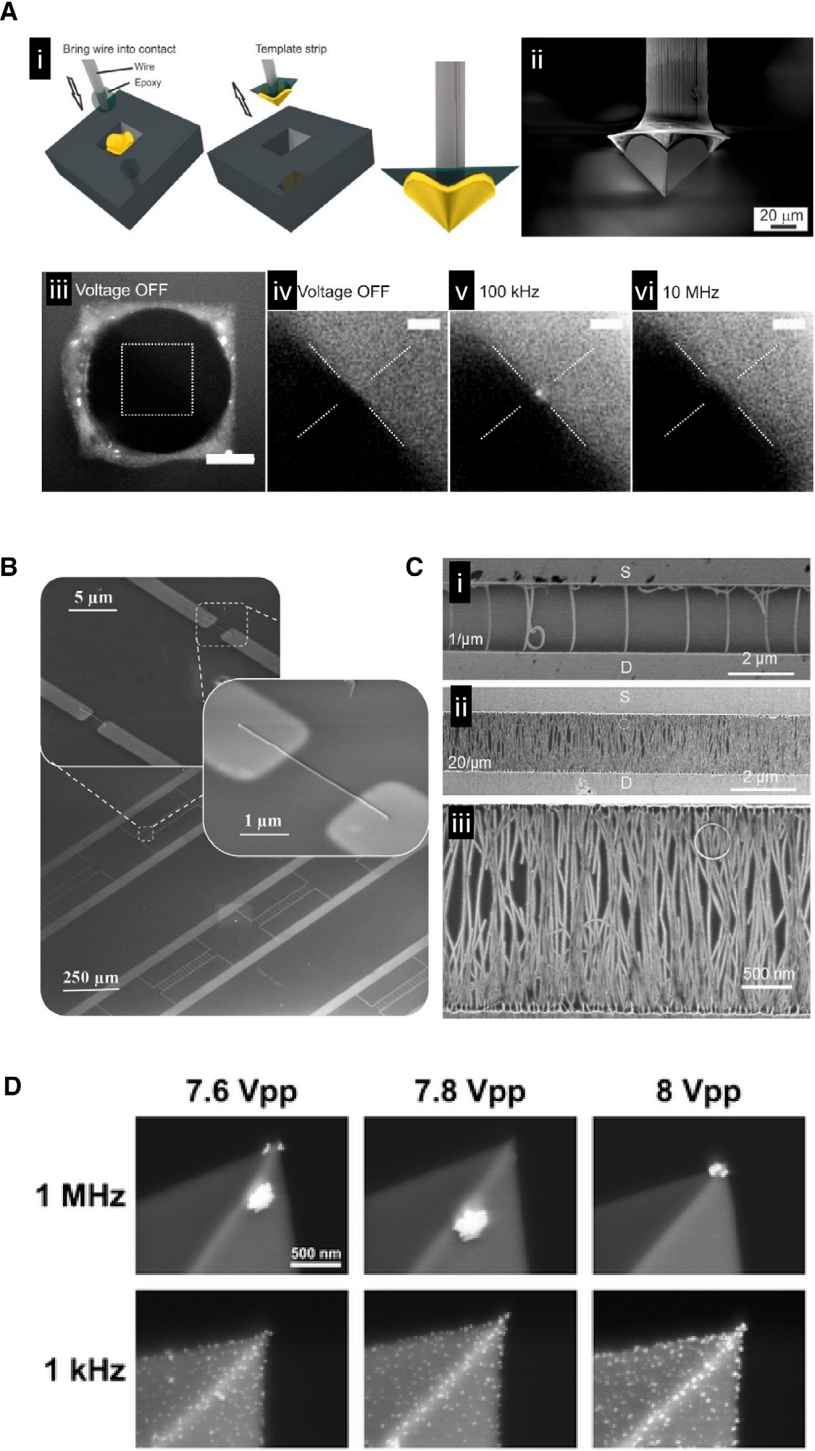
DEP‐based precision placement of nanoparticles. **(A)** Precision placement of a polystyrene nanobead at the tip of a pyramid electrode. **(i)** Manufacturing of the gold plated pyramid electrode. Conductive epoxy was used to connect a tungsten wire to a gold pyramid shaped electrode in a silicon mold. **(ii)** SEM image of the electrode. **(iii)** Florescence image focusing on the pyramid electrode base. Scale bar = 20 μm. **(iv–vi)** Fluorescence images focusing on the electrode apex. At 100 kHz, a single 190 nm polystyrene bead suspended in water was trapped at the tip of the pyramid. Scale bar = 5 μm. Reproduced with permission from the American Chemical Society: [109], Copyright 2014 (https://pubs.acs.org/doi/abs/10.1021/ph500091h, further permissions related to the material excerpted should be directed to the ACS). **(B)** DEP‐based alignment of nanowires between contact electrodes. These tilted SEM images show the precise DEP‐based localization between electrodes of a single semiconducting silicon nanowire. Adapted with permission from [121]. Copyright 2014, WILEY‐VCH. **(C)** These SEM images show the assembly of ultrahigh density aligned single‐walled carbon nanotubes (SWCNT) between DEP electrodes. Density of the SWCNTs was controlled by increasing their concentration in solution while holding the DEP parameters constant. **(i)** A density of ∼1 SWCNT/ μm. **(ii)** A density of 20 SWCNT/μm. **(iii)** Magnified image of panel (ii). Adapted with permission from [122]. Copyright 2011 American Chemical Society. **(D)** Patterning nanoparticles on atomic force microscopy tips using DEP. These SEM images show the spatial localization of 40 nm Ag nanoparticles on AFM tips using a range of different voltages and frequencies. The distance between the AFM tip and the counter electrode was ∼5 μm. Particles located near the tip can cause plasmonic enhancement for tip‐enhanced Raman spectroscopy measurements. Reprinted by permission from Springer Nature: [129], Copyright 2016.

Jose et al. integrated a gold pyramid electrode with an XYZ micro‐scanning stage to produce a mobile 3D DEP platform capable of isolating and concentrating nanostructures at the pyramid tip (Fig. 9A) [109]. Their “modified template‐stripping technique” used pre‐patterned silicon templates to fabricate gold tips with a 20 nm diameter and sub‐nanometer roughness. The precisely fabricated gold tips produced a highly localized electric field gradient for nanoparticle trapping at the tip's apex. Over an ITO glass substrate, a PDMS ring was patterned to hold the solution for DEP particle manipulation. The tip was suspended 70 μm from the ITO surface as an AC signal was applied between the ITO ground electrode and the metal tip to produce a DEP force within the liquid solution. The device showed the ability to selectively trap and repel single or multiple beads. Applying the signal for longer correlated with increased particle concentrations at the tip, improved stability of particles bound to the pyramid tip, and allowed for the capture of 190 nm polystyrene beads. Fixed voltages proved more effective at trapping larger beads than smaller beads for both multiple and single trapping tests. Single‐walled carbon nanotubes (SWCNTs) were also concentrated near the tip, with results confirmed using Raman spectroscopy. This platform has the possibility to simultaneously use DEP trapping to create metallic probe nanostructures which, along with the micromanipulation stage, may lead to developing novel scanning probe methods.

Lorenz et al. describes a new type of particle separation technique that involves DEP‐enhanced filtration allowing for the retention and release of sub‐micron particles [110]. Here, they used open porous microstructures consisting of ceramic filters/glass beads as a filter material sandwiched between electrodes. They were able to demonstrate the ability to switch between positive and negative DEP trapping by increasing the electric conductivity of the suspension. Particles were flowed into the filter, and would become trapped when the AC electric field was applied despite the constant fluid flow. When the AC electric field was turned off, the particles emerged from the filter. One of the benefits of using the porous material is that it allowed for a larger volume throughput than most traditional DEP devices. They showed selective trapping and recovery of 500 nm diameter polystyrene beads and 3 μm diameter graphite particles with fluid flow rates in the mL/min range. These high flow rates are important for future industrial applications.

### 4.2 Janus nanoparticles

A specialized subset of nanoparticles can be synthesized to have two halves that are made from or coated with different materials [111, 112]. This gives these Janus particles unique properties when interacting with an AC electric field compared to particles made from a single material because both halves of the same Janus particle will react differently [113].

Zhang et al. was able to show that polystyrene beads coated on just one hemisphere with gold or other dielectric materials drastically changed their behavior in the DEP field [114]. The precursor particles had a transition from positive DEP to negative DEP with increasing frequency but by coating the gold hemisphere of the particle with alkanethiol they were able to change the crossover frequency of the original particle and cause a reversal of behavior showing negative to positive DEP. Coating half of the sphere with a protein, such as fibronectin, also changed its DEP behavior [115].

Honegger et al. was able to show the ability of DEP to orient Janus particles made from half gold‐coated polystyrene spheres. By manipulating the frequency of the applied electric field, they were able to show preferential orientation of the gold‐coated side depending on the particle's positive or negative DEP response [116]. They were able to determine orientation based on the intensity of fluorescent signal emitted from the fluorescent polystyrene, which was occluded on the gold‐coated side. They were also able to demonstrate control over the frequency of rotation of these particles [117].

Boymelgreen et al. demonstrated that Janus particles can create their own DEP field when exposed to a uniform AC electric field [118]. This particle‐generated field can result in particle motion due to asymmetric electroosmotic flow around the particle due to its asymmetric design. This particle‐generated field can also result in DEP. Smaller particles can undergo positive or negative DEP toward or away from the gold‐coated side depending on the frequency of the applied uniform electric field. Particle‐generated DEP has also been shown to be able to attract cellular organelles and could be used for organelle transport and sorting applications [119].

### 4.3 Drug delivery vehicles

Drug delivery vehicles are designed to encapsulate or otherwise contain therapeutic compounds and carry them through circulation to the target tissue region where they preferentially deliver their payload. This helps to concentrate drugs in the tissue where they are needed and reduces side effects from non‐target tissues being exposed to the drugs. The circulation time of these particles is a critical aspect of their performance and can be influenced by several factors. Understanding the changes that occur to the surface of the particles while in circulation is an important aspect to improve their circulation time. Here DEP has a unique ability to preferentially recover the drug delivery vehicles from circulation to enable study of their surfaces.

Ibsen et al. used high conductance DEP for drug delivery nanoparticle recovery from blood plasma [120]. A suspension of fluorescently labeled, silica shell nanoparticles, empty liposomes, filled liposomes, or polymer‐based drug delivery vehicles in 1× PBS was spiked into human plasma at a ratio of 10–90 μL, respectively, and were recovered using a 15 kHz AC signal. Fluorescence from nanoparticles accumulated at the electrode confirmed collection after 7 min of signal application. The collected particles were washed, recovered, and examined using scanning electron microscopy to visually analyze their surfaces for protein deposition due to the plasma exposure.

### 4.4 DEP‐based nano‐/microfabrication

Due to their small size, nanoparticles are extremely difficult to physically manipulate and orient in a manner required to fabricate larger structures. The ability to manipulate nanoparticles and move them to a desired location and in a desired pattern is a unique aspect of DEP technology that is well suited for nanofabrication. The choice of the DEP technique and electrode geometry can greatly influence the nature of the assembled structures.

#### 4.4.1 Nanotubes and nanowires

Nanotubes are a versatile class of nanoparticles that have varied applications. Spatially manipulating them is a challenge due to their small size and high aspect ratio. DEP can contribute to this field by preferentially creating forces on these particles to move and orient them in ways that physical manipulation cannot achieve.

Collet et al. developed a capillary‐assisted DEP technique to assemble single nanowires onto metal contacts (Fig. **9B**) [121]. This method focused on precisely aligning individual nanowires relative to the device geometry. A solution of nanowires was introduced to the metal contact region and convective flow was generated in the solution while the liquid evaporated which resulted in an increase in nanowire concentration at the liquid–substrate interface. This allowed for DEP trapping and orientation of a single nanowire. Finally, capillary forces held the nanowires in place as the liquid solution evaporated. An applied AC signal of 50  kHz to a solution containing silicon nanowires, ≈50 nm in diameter and 5 μm long, resulted in a proper nanowire alignment in 81% of specified locations. A mixed solution of silicon and indium arsenide nanowires was used to create alternating arrays of aligned nanowires by switching the applied signal frequency from 50 to 500 kHz for alignment of silicon and indium arsenide, respectively.

Shekhar et al. developed a DEP‐based technique to fabricate aligned arrays of high density single‐walled carbon nanotubes (SWCNTs) on metal contacts, separated by a specified length (Fig. **9C**) [122]. This process was optimized such that control of the number of SWCNTs along the metal was achieved through the concentration of the nanotube solution. A maximum density of 30 SWCNT/μm was demonstrated, which was the same density achieved using chemical vapor deposition, which required a two‐step growth process followed by transfer printing to produce aligned arrays. Here, a nanotube concentration of 6.8 μg/mL was used to achieve the maximum reported density, and further increase in concentration led to multilayer nanotube films. The length of the channels the nanotubes were aligned across varied from 2 to 10 μm with width varying from 100 to 1000 μm. Interestingly, the quality and density of the SWCNT alignment did not vary with length nor did the linear density of nanotubes depend on channel width when all DEP parameters were held constant.

Rabbani et al. used an iDEP microfluidic platform to enable DEP‐trapping of surface‐modified SWCNTs and manipulated their orientation within the chip's insulator structure [123]. They designed a device containing an array of 15 μm insulating posts within a 1.5 cm microchannel, which was used over a range of voltages. SWCNT capture was observed using IR fluorescence. Surfactant and single‐stranded DNA‐wrapped SWCNTs were prepared by incubating the SWCNTs in either sodium deoxycholate or T30 single‐stranded DNA followed by probe sonication. DEP‐trapping was reported for the DNA coated samples sonicated for 90 min and the sodium deoxycholate coated samples sonicated for 20 and 60 min. Smaller SWCNTS required greater voltages to achieve DEP trapping. They showed that by changing conditions they could trap the nanotubes in positive DEP to get a vertical connection between the posts and in negative DEP to orient the nanotubes horizontally between posts.

Constantinou et al. used a 3D well electrode DEP device to selectively isolate silicon nanowires with the desired electrical properties from other nanowires grown in the same solution [124]. The 3D‐DEP DEPtech device contained an array of wells with copper electrodes and polyimide structures that separated the electrodes and defined the sides of the wells. The electrode spacing was 150 μm and the well height was 1 mm. DEP forces were analyzed by detecting variations in the intensity of transmitted light as the nanowires moved between trapped and untrapped particle states in each well. Silicon nanowires were grown by a vapor‐liquid‐solid (VLS) mechanism using a gold catalyst and a silicon <111> substrate. The nanowires were 75 nm in diameter and 22 μm in length. The desired nanowires were aligned on metal contacts with an AC signal to produce field effect transistors (FET). Between 50 and 230 nanowires bridged across the 10 μm channels of the transistor. The nanowires aligned using 10 MHz and 20 MHz signals showed a 100‐fold increase in current per nanowire, a 50% decreased subthreshold swing, and approximately a 100 times greater mobility relative to nanowires collected at lower frequencies.

Seichepine et al. used a liquid‐coupled floating‐electrode DEP method to integrate 1024 carbon nanotubes (CNT) onto a complementary metal oxide semiconductor platform without the need for a transfer step [125]. 80% of the sensor locations contained one‐to‐five CNTs after DEP alignment, and each CNT sensor was individually addressable. Using a 300 kHz signal to effect positive DEP forces, CNT deposition was tracked over time using a voltage‐contrast scanning electron microscope (VC‐SEM). After 45 min, nearly 100% of electrode pairs were connected by DEP‐deposited CNTs. For sufficiently long deposition times, 100% of DEP sites were filled with dense CNT assemblies. SEM images taken at 20, 30, and 40 min of DEP assembly indicated that CNTs were attracted to the edges of grounded electrodes.

Freer et al. developed a DEP‐based process for high yield silicon nanowire alignment on patterned electrodes [126]. To position single nanowires, a nanowire solution was flowed through a microchannel with electrode‐patterned walls. The net force on the nanowires was the sum of the positive DEP, hydrodynamic, and electrostatic double‐layer forces. Nanowires aligned parallel to the electric field due to torque derived from their polarization and the alignment process was reversible below the point of high voltage adhesion. The flow rate and voltage was optimized to achieve consistent nanowire assembly for single nanowire configurations and stable double nanowires on individual electrodes. The final array contained eight sites, each with “multi‐finger” electrode structures assembled with single nanowires. They achieved 98.52% of the 96 total sites being populated by single nanowires. The group determined a “power law exponent of ≈2” to describe the relationship between flow rate and applied voltage, which was used to identify the ideal range for achieving single nanowire alignments. All experiments were observed using deep ultraviolet imaging techniques.

#### 4.4.2 Particle and droplet assemblies

Tang et al. utilized a DEP microfluidic platform to fabricate 3D Galinstan (a liquid metal made primarily from gallium, indium, and tin) microstructures that enhanced the device's DEP trapping capabilities [127]. The device was composed of a glass substrate with an array of 80 μm diameter, planar, chromium/gold circular microelectrodes. First, a liquid droplet of Galinstan was suspended in deionized water and broken into Galinstan microdroplets (2 μm average) via ultrasonic agitation. A native oxide formed on the surface of the droplets. A microfluidic device isolated the droplets out of suspension within 10 s by positive DEP using a 20 MHz AC signal. A uniform distribution of droplets along the electrodes was achieved by adjusting the flow rate and voltage over time. The native oxide on the microdroplet surfaces was then removed by flowing in a 0.1 M NaOH solution, which was coupled with an increase in voltage thereby promoting the formation of larger droplets. Galinstan subsequently wetted the microelectrode surface to form a hemisphere with a small contact angle. Lastly, a higher flow rate was used to remove unmerged microdroplets. The heights of the Galinstan hemispheres ranged from 25 to 40 μm, as measured by curing PDMS over the microstructures. The height could be changed by varying the immobilization time. This process was repeated for smaller planar electrodes, down to a minimum diameter of 20 μm. The microstructured Galinstan enhanced the DEP device's ability to trap 80 nm WO_3_ particles by 40% compared to planar electrodes alone.

Cha et al. used DEP to cold weld 20 nm gold nanoparticles together into nanoribbons on a mica substrate [128]. The results were imaged using high‐resolution transmission electron microscopy and atomic force microscopy (AFM). The DEP force led to nanoparticle accumulation at the edges of the nanostructure while coulombic forces led to nanoparticle chaining, where the cold‐welding process was initiated by forces between the particles and the mica substrate. Low nanoparticle concentrations failed to begin the welding process due to weak coulombic attractions, whereas high nanoparticle concentrations resulted in predominantly radial growth with no elongated structures formed. AFM images confirmed coulombic aggregation of “nanoleafs,” while COMSOL analysis considered the effects from electrode geometry. Nanoribbons typically formed orthogonally to the electrodes, and particle formation was most easily controlled via the signal frequency. The resulting nanoribbon's dimensions varied between 1.6 and 4.7 nm in height, 11 and 28 nm in width, and 0.4 and 1 μm in length, and the group found that electric field magnitude had little influence on agglomeration but did influence alignment.

Leiterer et al. used a DEP micromanipulator device to attach silver nanoparticles onto heavily doped silicon AFM tips with nanoscale precision and with no chemical modification (Fig. 9D) [129]. 40 nm silver particles, in a 2×10^11^ particles/mL suspension, were tested from 1 kHz to 10 MHz, and it was shown that localization to the tip depended on frequency, as confirmed using SEM and TEM imaging. At 1 kHz, DEP was not the dominant factor in deposition and instead unreacted Ag ions in the supernatant were electrochemically growing on the AFM tip, yielding increased growth with both time and voltage. Particle loading onto the tip remained relatively constant with voltage and proportional to particle density at 1 MHz, with a string‐of‐pearls effect developing at high concentrations. Tip‐enhanced Raman spectroscopy was demonstrated with these tips prepared by DEP particle loading at 1 MHz.

Rozynek et al. developed a hybrid DEP‐capillary technique to fabricate stable conductive particle chains using a macroscopic needle electrode and a liquid suspension of particles, consisting of 60 μm silver coated silica shells in silicone oil [130]. A 20 kHz signal was applied after placing the electrode tip in the solution, and the electrode was drawn up at a rate of 1 mm per second, leading to the formation of particle chains as long as 3 cm, formed in under 1 min. Pull rates higher than the 1 μm/s were shown to cause kinks in the chains, and results were limited to micron‐sized particles. Chains were deposited on surfaces by removing the AC signal. This platform successfully deposited 25 μm, 55 μm, and 100 μm particles and developed C, S, and L shaped patterns.

Manipulating objects below 1 μm in diameter with any sort of precision is an immensely challenging task. These manipulations include separating nanoparticles for manufacturing applications where it is important to collect nanoparticles with the desired properties from heterogeneous synthesis mixtures. It is also important for environmental studies where quick and field deployable devices for the detection of nanoparticle pollution and contaminates in soil and water, and biomarkers from tissues is an increasingly unmet need. Nanofabrication is also an application where the ability to create forces on the particles to spatially manipulate them without the need to physically contact them is a great benefit, both from the consideration of what can be practically done to nanoparticles in terms of placement and orientation and also from scalability where many nanoparticles need to be manipulated at once. The range of applications described in this section demonstrate the important role that the DEP force can have, as defined spatially by clever and versatile electrode and chip designs. As in the previous sections, DEP has been demonstrated work on a wide range of targets, in this case different nanoparticle types, ranging from spherical nanobeads to high aspect ratio nanowires. Both iDEP and direct electrode‐based DEP can be successfully applied to these applications.

## 5 Discussion and Conclusion

The last 10 years has seen exciting new developments in the field of DEP, a large portion of which have been focused on biological applications. The ability to selectively create forces on small objects ranging from molecules, to nanoparticles, to entire blood cells enables the manipulation of individual particles in a size range that is inherently challenging to spatially control with precision. As described above, the ability to hold, move and examine proteins, bacteria, yeast, exosomes, DNA, stem cells, cancer cells, and blood cells opens up unique opportunities to ask biological questions and find answers that are unattainable using traditional methods. The challenges of pursuing questions in the fields of biology and medicine inherently reside with studying the behaviors of microscopic units and there is a great need to manipulate individual cells, biologically derived nanoparticles, and molecules. DEP is a technique that can deliver those abilities and opens the door for the development of new analytical equipment and techniques that can be applied to predict cellular differentiation and behavior, diagnosis disease, detect pathogens, and quickly ascertain the effectiveness of different therapeutics.

Microfabrication is also an exciting field that DEP can significantly contribute to as well. Inherent in this field is the need to manipulate extremely small particles in precise ways. DEP offers an ability to move and orient individual nanoparticles, even those with high aspect ratios, into predefined locations in ways that other techniques cannot match.

The observed rise in patent applications dealing with DEP technology over the last 10 years indicates that DEP is beginning to enter an era of commercialization. Four companies mentioned in this review, Biological Dynamics, DEPtech, Cbio.io, and Menarini‐Silicon Biosystems, have already been founded on DEP technology.

The sheer diversity of ideas and approaches shown by the authors of the papers highlighted here over the last 10 years indicates that DEP has an exciting decade ahead. Advances that will undoubtedly occur in chemistry, material science, and engineering will help expand the already impressive capabilities of existing DEP devices and enable the creation of new ones. These changes and improvements will further push DEP technologies toward commercialization, industrial applications, and continue the increasing trend toward biological and clinical applications.

*This project was supported by funding (Project 68181‐935‐000) from the Cancer Early Detection Advanced Research Center at Oregon Health and Science University's Knight Cancer Institute*.

*Dr. Michael Heller is a member of the scientific advisory board for the company Biological Dynamics. Dr. Daniel Heineck was previously employed by Biological Dynamics*.
